# Interferon-induced transmembrane protein-1 competitively blocks Ephrin receptor A2-mediated Epstein–Barr virus entry into epithelial cells

**DOI:** 10.1038/s41564-024-01659-0

**Published:** 2024-04-22

**Authors:** Yinggui Yang, Tengteng Ding, Ying Cong, Xiaomin Luo, Changlin Liu, Ting Gong, Min Zhao, Xichun Zheng, Chenglin Li, Yuanbin Zhang, Jiayi Zhou, Chuping Ni, Xueyu Zhang, Ziliang Ji, Tao Wu, Shaodong Yang, Qingchun Zhou, Dinglan Wu, Xinqi Gong, Qingyou Zheng, Xin Li

**Affiliations:** 1 Shenzhen Key Laboratory of Viral Oncology, Department of Urology, and Clinical Innovation and Research Centre (CIRC), Shenzhen Hospital of Southern Medical University, Shenzhen, Guangdong China; 2 The Third School of Clinical Medicine, Southern Medical University, Guangzhou, Guangdong China; 3 PANACRO(Hefei) Pharmaceutical Technology Co. Ltd., Hefei, China; 4 Mathematical Intelligence Application LAB, Institute for Mathematical Sciences, Renmin University of China, Beijing, China

**Keywords:** Virus-host interactions, Viral infection

## Abstract

Epstein–Barr virus (EBV) can infect both B cells and epithelial cells (ECs), causing diseases such as mononucleosis and cancer. It enters ECs via Ephrin receptor A2 (EphA2). The function of interferon-induced transmembrane protein-1 (IFITM1) in EBV infection of ECs remains elusive. Here we report that IFITM1 inhibits EphA2-mediated EBV entry into ECs. RNA-sequencing and clinical sample analysis show reduced IFITM1 in EBV-positive ECs and a negative correlation between IFITM1 level and EBV copy number. IFITM1 depletion increases EBV infection and vice versa. Exogenous soluble IFITM1 effectively prevents EBV infection in vitro and in vivo. Furthermore, three-dimensional structure prediction and site-directed mutagenesis demonstrate that IFITM1 interacts with EphA2 via its two specific residues, competitively blocking EphA2 binding to EBV glycoproteins. Finally, YTHDF3, an m^6^A reader, suppresses IFITM1 via degradation-related DEAD-box protein 5 (DDX5). Thus, this study underscores IFITM1’s crucial role in blocking EphA2-mediated EBV entry into ECs, indicating its potential in preventing EBV infection.

## Abstract (web summary)

IFITM1 can compete with EBV glycoproteins for EphA2 binding and prevent virus entry into epithelial cells, in vitro and in vivo. YTHDF3 suppresses IFITM1 via the degradation-related protein DDX5.

## Main

Epstein–Barr virus (EBV) is the first oncogenic herpesvirus that targets epithelial cells (ECs) and B lymphocytes^1^. It infects ~95% of the population worldwide^2^ and is associated with a spectrum of severe diseases, especially mononucleosis and various forms of cancer, including nasopharyngeal carcinoma (NPC), gastric cancer, colorectal cancer and B cell lymphoma^3–6^. Effective prevention of EBV infection is a crucial public health issue.

During EBV infection, viral glycoproteins collaborate with host envelope proteins to enable membrane fusion and EBV entry into target host cells^7^. Core to this process is the glycoprotein B (gB) homotrimer and the glycoprotein H/L (gH/gL) heterodimer, facilitating fusion in ECs and B lymphocytes^8–11^. Over the past several decades, extensive research has been conducted on B cell receptors involved in EBV entry^11–15^. As to ECs, integrins were initially identified to be the primary receptors for EBV entry^16,17^, but they were later confirmed to be accessory receptors^18,19^. More recently, following the discovery of Neuropilin 1 (NRP1) and Non-muscle myosin IIA (NMHC-IIA, also known as MYH9)^20,21^, Ephrin receptor A2 (EphA2) has been reported as a crucial EC receptor for EBV entry^18,19^. EphA2 confers the susceptibility of ECs to EBV by binding to viral gH/gL and gB to facilitate the internalization and fusion of EBV^19^. Nonetheless, it was surprising to find that EphA2 was highly expressed in ECs with low susceptibility to EBV^22^, suggesting that additional unknown factors influenced host susceptibility to EBV infection.

Interferon-induced transmembrane protein-1 (IFITM1), a vital IFITM family member, plays a crucial role in host defence against various viruses^23–25^ by suppressing viral entry and replication^26,27^. Although IFITM1 has been demonstrated to hinder some viruses^28–30^, its effects on EBV infection appear to be insufficiently studied. IFITM1 has been reported to contribute to EBV infection in BJAB (human B lymphoma cells) and HMVEC-d (human microvascular endothelial cells)^31,32^, yet their underlying mechanism remains unclear. Currently, there appears to be no relevant research on the involvement of IFITM1 in EBV infection of ECs. Further research is necessary to elucidate IFITM1’s function in EBV infection in ECs and to develop effective early-intervention strategies.

N6-methyladenosine (m^6^A) modification is crucial in the regulation of gene expression by affecting mRNA stability, splicing, localization and translation efficiency^33^. YTH-domain family protein 3 (YTHDF3), an m^6^A reader, has been found to affect translation and degradation, negatively impacting interferon-mediated antiviral immunity^34^, but its role in regulating IFITM1 is not yet understood. Further investigation is required to explore the relationship between YTHDF3 and IFITM1 during EBV infection of ECs.

Thus, this study aims to explore the role of IFITM1 in EBV infection of ECs, focusing on its effect on the interaction between host EphA2 and viral gH/gL or gB. In addition, we seek to understand how IFITM1 influences EBV infection through the perspective of m^6^A modification. This research may reveal additional insights into the complex process of EBV infection in ECs and potential strategies for preventing EBV infection.

## Results

### IFITM1 negatively correlates with EBV infection in ECs

**Extended Data Fig. 1:**
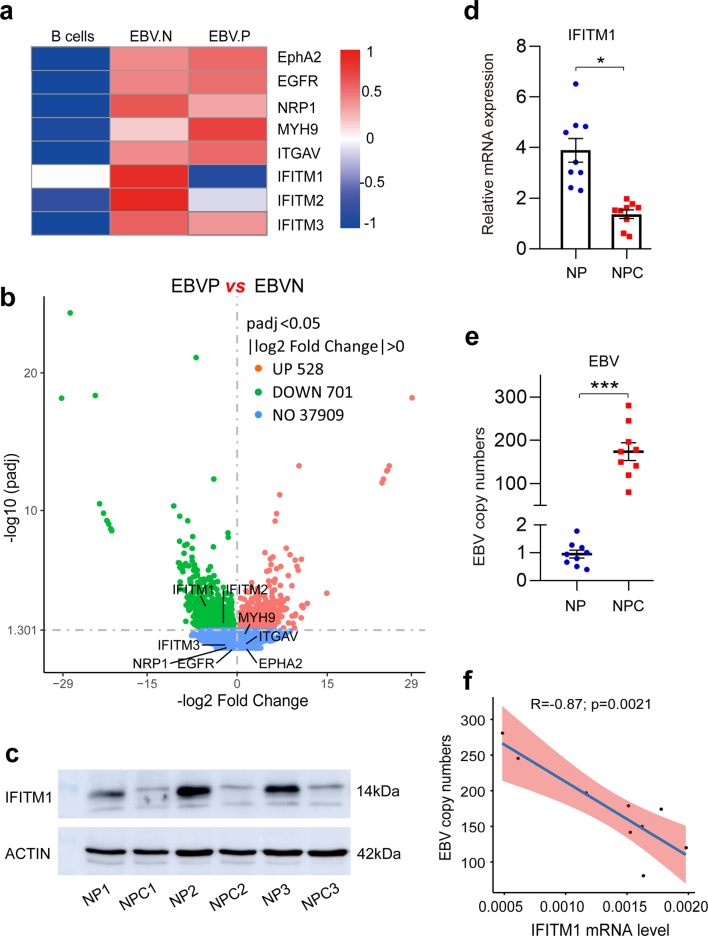
IFITM1 negatively correlates with EBV infection in ECs. (**a**) Heatmap of the mRNA expression levels (FPKM) of IFITMs and the reported epithelial cell receptors in B cells, EBV-negative (EBV.N) cells and EBV-positive (EBV.P) cells, analyzed by RNA sequencing. Data were plotted as the log_10_ of transformed cells for ease of comparison and visualization. (**b**) Volcano plot of the differentially expressed genes in the EBV-negative (EBVN) and EBV-positive (EBVP) groups, with upregulated genes shown in red, downregulated genes in green, and no differentially expressed genes in blue. The horizontal-axis represents −log_2_ (fold-change), and vertical-axis represents −log_10_ (*p*-value). n = 2 independent experiments; *p < 0.05, two-tailed *t*-test. (**c**) The Western blot analysis of tissue lysates from three NPC and three nasopharynx (NP) samples for IFITM1 protein expression using actin as a loading control, n = 3 pairs. (**d**) The mRNA level of IFITM1 in nine NPC and nine NP samples was assayed by RT-qPCR, and B_2_M was used as a reference gene, n = 9 pairs. (**e**) The EBV copy numbers in nine NPC and nine NP samples were also assayed by TaqMan-qPCR using an EBV detection kit, n = 9 pairs. Data are presented as mean values ± s.e.m., n = 9 pairs, *p < 0.05, **p < 0.01, ***p < 0.001 (**d, e**), two-tailed t-test. (**f**) A correlation analysis was performed between the relative IFITM1 mRNA expression and EBV copy numbers in nine NPC samples (horizontal axis: relative IFITM1 mRNA levels; vertical axis: relative EBV copy numbers). The black dot denotes scattered samples. Data are presented as mean values ± s.e.m., n = 9, r = −0.87, *p* = 0.0021 (**f**), two-tailed t-test. Source data

To elucidate the mRNA expression profiles of essential epithelial-cell receptors and IFITMs, we conducted RNA-sequencing on B cells (Daudi), EBV-negative ECs (EBV.N, including NP69, NP460, HK1, HEK293) and EBV-positive epithelial (tumour) cells (EBV.P, including NP460-EBV, HK1-EBV, C666-1). We observed that EphA2, an EBV entry receptor previously identified in ECs, exhibited higher expression levels in ECs than in B cells (Extended Data Fig. 1a). This is consistent with the notion that EphA2 expression is specific to ECs and absent in B cells^19^. A similar trend was seen in other receptors such as MYH9, integrin alpha V (ITGAV), NRP1 and epidermal growth factor receptor (EGFR) in ECs. These receptors were upregulated in EBV.P cells compared with EBV.N cells (Extended Data Fig. 1a). Conversely, IFITMs showed lower expression in EBV.P cells than in EBV.N cells, with IFITM1 being the most significantly downregulated (−log_2_ fold change = −4.48) (Extended Data Fig. 1a,b).

To further explore the relationship between IFITM1 and EBV infection, we also detected IFITM1 level and EBV copy number in NPC tissues and non-cancerous nasopharynx (NP) tissues. As expected, IFITM1 was significantly lower in NPC tissues than in NP tissues (Extended Data Fig. 1c,d), while EBV copy number was barely detectable in NP tissues but readily detected in NPC tissues (Extended Data Fig. 1e), aligning with previous findings^35–38^. A strong negative correlation (*R* = − 0.87, *P* = 0.0021) was found between IFITM1 level and EBV copy number (Extended Data Fig. 1f).

Collectively, these data suggest a significant negative correlation between IFITM1 and EBV infection in ECs. Given IFITM1’s role as a cell surface barrier, we subsequently focused on its function in EBV infection in ECs.

### IFITM1 inhibits EBV infection in ECs

**Extended Data Fig. 2:**
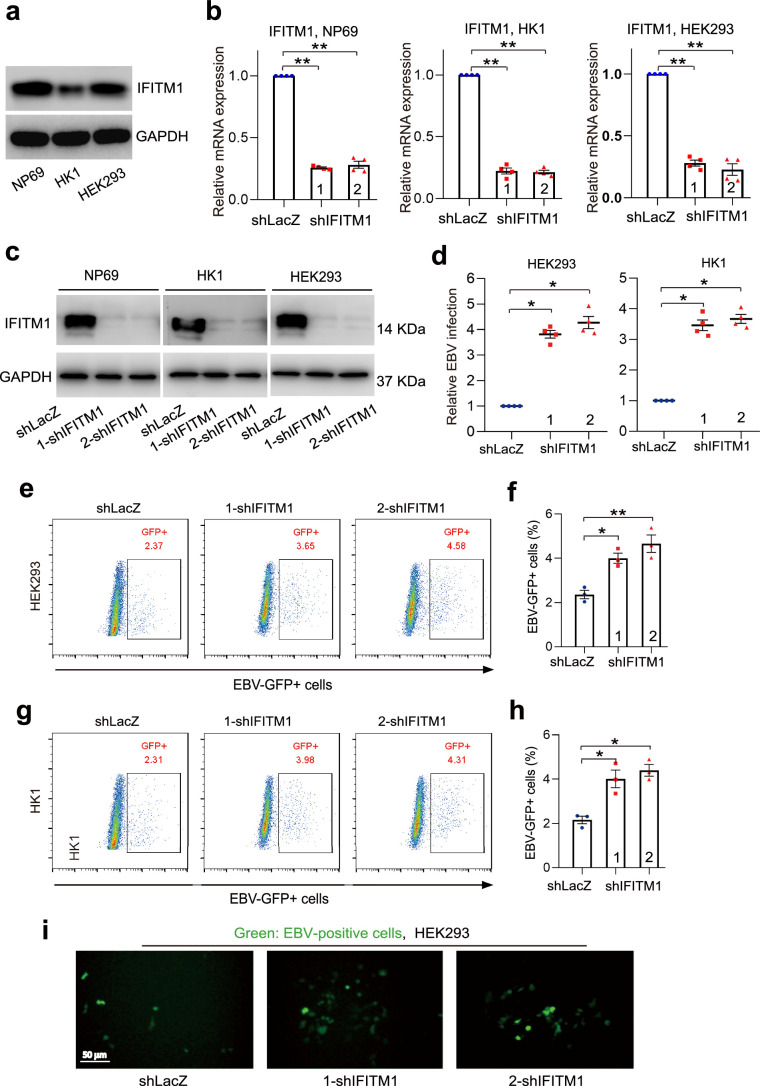
Knockdown IFITM1 facilitates EBV infection in ECs. (**a**) NP69, HK1, and HEK293 cell lines were collected, and immunoblotting assayed expression of IFITM1 at the protein level. (**b-c**) Knockdown of IFITM1 in NP69, HK1, and HEK293 cells by infection with lentiviruses encoding shIFITM1 (1-shIFITM1 and 2-shIFITM1). shLacZ-transfected cells were included as a control. (**b**) Knockdown efficiency of IFITM1 at the mRNA level. Bars show the relative IFITM1 mRNA level detected by RT-qPCR and data are presented relative to B_2_M (2^˗ΔCt^). (**c**) Knockdown efficiency of IFITM1 at the protein level. (**d**) HEK293 and HK-1 cell lines were incubated with cell-free EBV-GFP for 3 hours, and EBV copy numbers were then measured by TaqMan-qPCR. (**e-h**) After 72 hours, flow cytometric analyses were performed to show the percentage of EBV-GFP-positive cells. Representative images of cells infected with EBV-GFP were recorded for HEK293 cells (**i**). Data are presented as mean values ± s.e.m., n ≥ 3 independent experiments, *p < 0.05, **p < 0.01, two-tailed *t*-test (b, d-h). Source data

To further explore IFITM1’s role in EBV infection, we conducted a series of experiments in various ECs, including NPC-derived ECs (HK1) and normal ECs (NP69, HEK293). Western blotting showed that HK1 displayed a relatively low level of IFITM1, whereas NP69 and HEK293 exhibited relatively high levels of IFITM1 (Extended Data Fig. 2a).

**Fig. 1:**
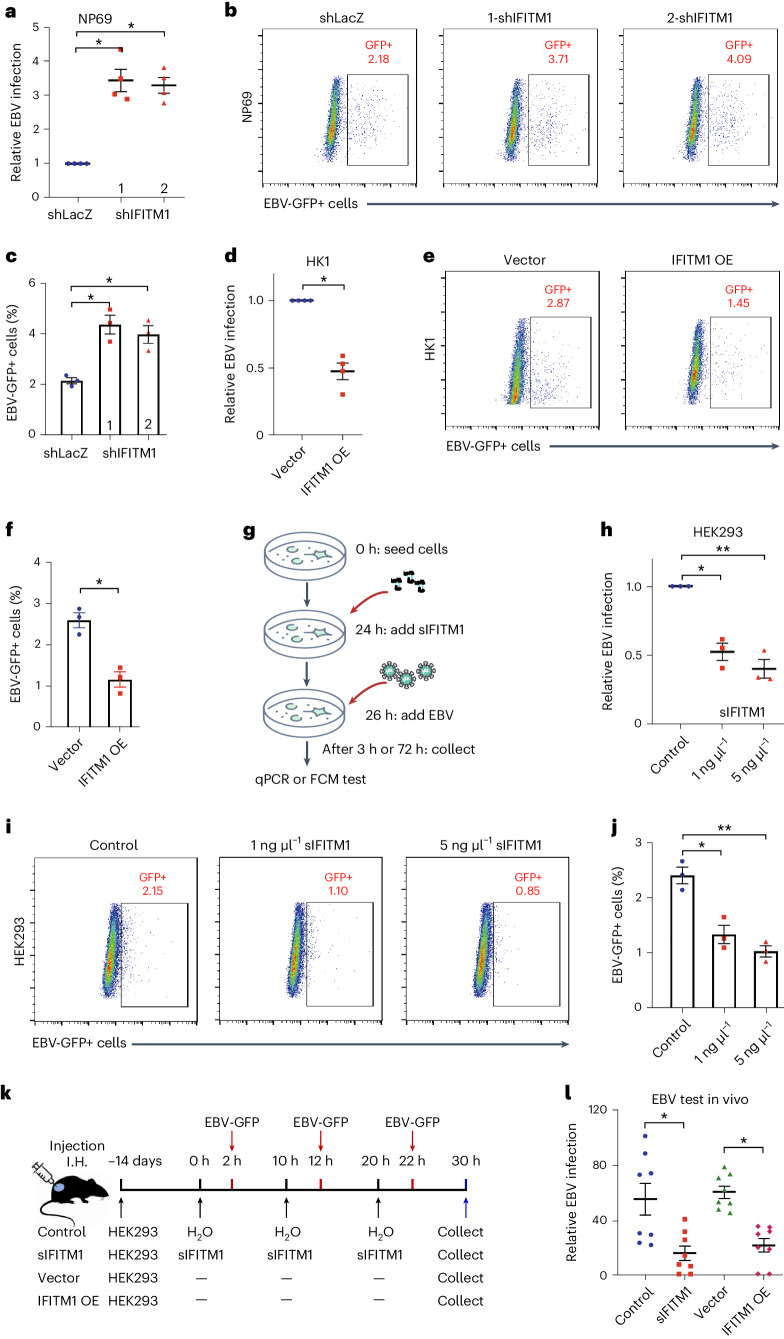
IFITM1 inhibits EBV infection in vitro and in vivo*.* **a**, NP69 cell lines (from Extended Data Fig. 2b,c) were incubated with cell-free EBV-GFP for 3 h, after which EBV copy numbers were measured by *Taq*Man−qPCR. **b**,**c**, Flow cytometric analyses (**b**) were conducted after 72 h to determine the percentage (**c**) of EBV-GFP-positive cells. The numbers 1 and 2 represent two different sequences of shIFITM1, which can be referred to as 1-shIFITM1 and 2-shIFITM1, respectively. **d**, HK1 cell lines (from Extended Data Fig. 3a,b) were incubated with cell-free EBV-GFP for 3 h, followed by EBV copy number detection using *Taq*Man−qPCR. **e**,**f**, After 72 h, flow cytometric analyses (**e**) were performed to assess the percentage (**f**) of EBV-GFP-positive cells. The results are presented as mean ± s.e.m. of at least 3 biological replicates. *P < 0.05 (*n* ≥ 3, **a**,**c**,**d**,**f**), two-tailed *t*-test. **g**–**j**, Analysis of the effects of sIFITM1 in vitro was performed as follows: **g**, A flow chart showing exposure of HEK293 cells to EBV-GFP for 3 h, with sIFITM1 being added 2 h in advance at different concentrations (0, 1 and 5 ng µl^−1^). **h**, EBV copy numbers were measured by *Taq*Man−qPCR. **i**,**j**, At 72 h after EBV exposure, flow cytometric analyses (**i**) were performed to show the percentage (**j**) of EBV-GFP-positive cells. Data are presented as mean ± s.e.m., *n* = 3 independent experiments, *P < 0.05, **P < 0.01, two-tailed *t*-test (**h**,**j**). **k**, Nude mice were xenografted with HEK293 to form epithelial-cell clusters, followed by injection of sIFITM1 and EBV-GFP at set intervals. A control group was included in which double-distilled H_2_O was injected at the corresponding intervals. I.H., hypodermic injection. **l**, Equal amounts of total DNA were obtained from the tumour-like cell clusters to detect the EBV copy numbers by *Taq*Man−qPCR for the four groups. For each group, *n* = 8 and ‘−’ indicates no treatment. Results are expressed as mean ± s.e.m. *P < 0.05, two-tailed *t*-test. Source data

**Fig. 2:**
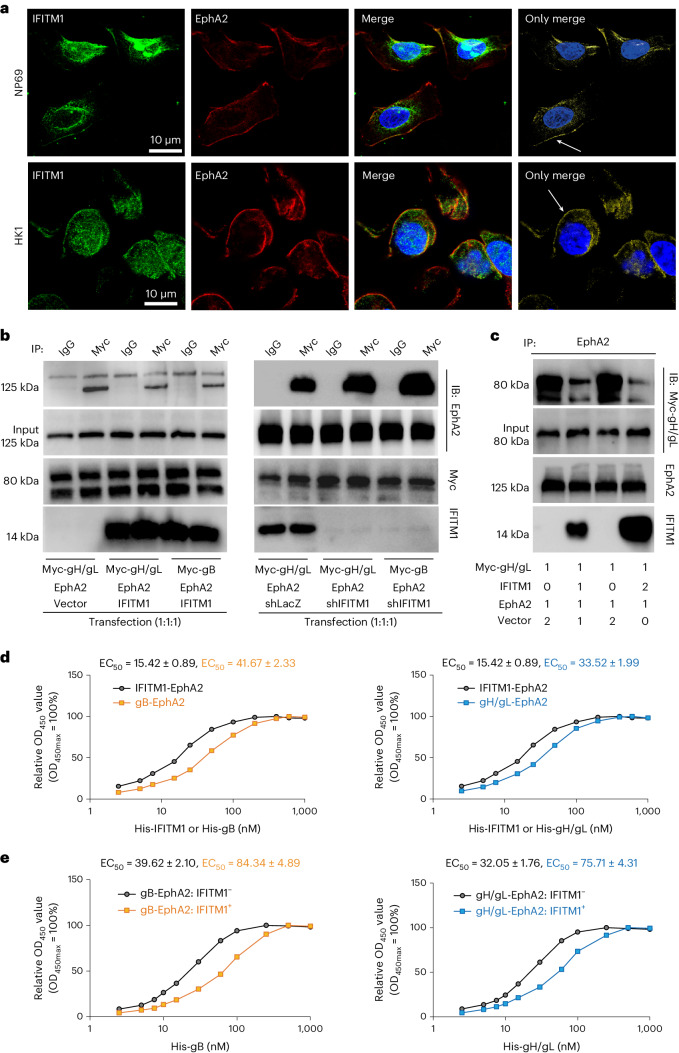
IFITM1 competes with EBV gH/gL and gB for binding of EphA2. **a**, IF assays showed the co-localization of IFITM1 with EphA2 in NP69 and HK1 cells. IFITM1 was stained by Alexa fluor 488 (green) and EphA2 by Alexa fluor 647 (red). The co-localization of IFITM1 and EphA2 was visualized as a yellow signal (white arrows). **b**, Co-IP assays showed that IFITM1 influenced the binding of EphA2 to gH/gL or gB. Three combinations of plasmids were designed and three different plasmids were transfected into each group at a ratio of 1:1:1. The reference group was transfected with Myc-gH/gL, EphA2 and an empty vector, while the comparison groups were transfected with gH/gL, EphA2 and IFITM1, or gB, EphA2 and IFITM1 (on the left). Simultaneously, the reference group was also transfected with Myc-gH/gL, EphA2 and shLacZ, while the comparison groups were transfected with gH/gL, EphA2 and shIFITM1, or gB, EphA2 and shIFITM1 (on the right). Cell lysates were immunoprecipitated with anti-Myc antibody, followed by immunoblotting (IB) analysis with anti-EphA2, Myc and IFITM1 antibody. **c**, Co-IP assays showed that the effect of IFITM1 on binding of EphA2 to gH/gL was dose dependent. After 48 h transfection, cell lysates were collected and then immunoprecipitated with an anti-EphA2 antibody, followed by immunoblotting analysis with anti-Myc, EphA2 and IFITM1 antibodies. The numbers below each band indicate the transfection dosage. **d**, ELISA showed the affinity constant of the interaction between EphA2 and IFITM1, gH/gL or gB. **e**, ELISA showed the affinity constant of the interaction between EphA2 and gH/gL or gB with/without IFITM1. All results were obtained from at least 3 biological replicates. Source data

**Extended Data Fig. 3:**
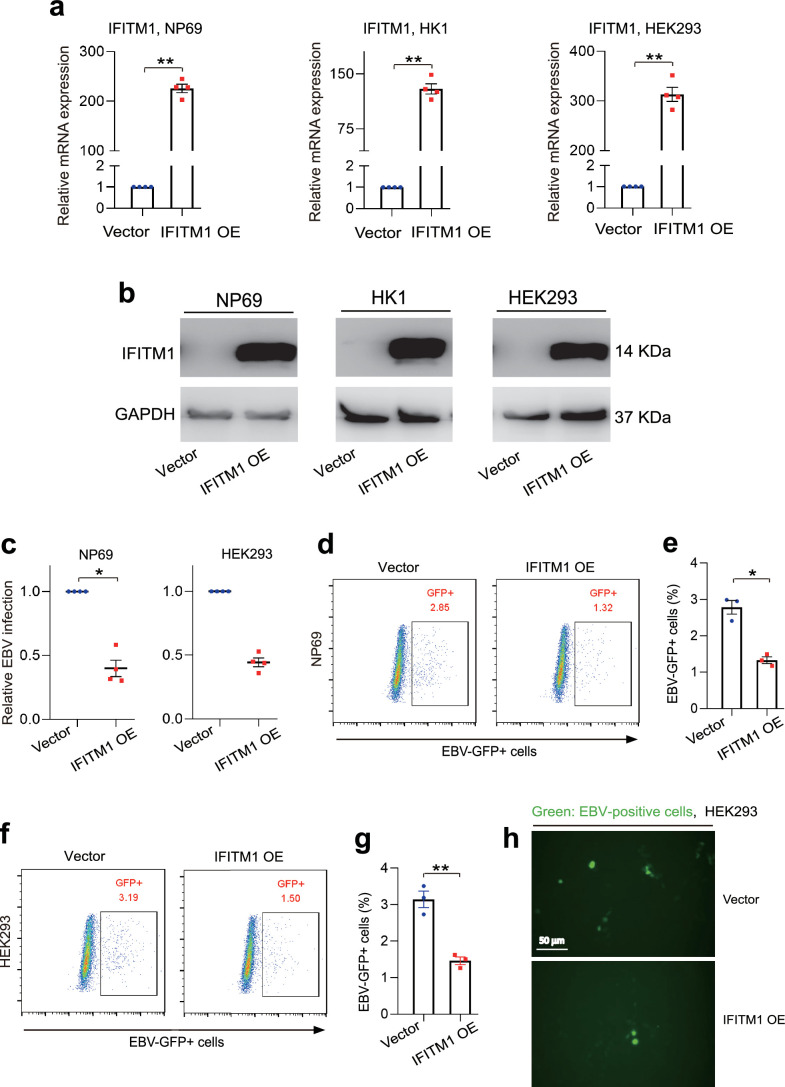
Overexpression IFITM1 inhibits EBV infection in ECs. (**a**) The efficiency of overexpression of IFITM1 at the mRNA level. Bars show the relative IFITM1 mRNA level detected by RT-qPCR and data are presented relative to B_2_M (2^˗ΔCt^) and are expressed as the mean ± s.e.m. (n = 3 independent experiments, ***p < 0.001), two-tailed *t*-test. (**b**) Efficiency of overexpression of IFITM1 at the protein level. GAPDH was used as a control to indicate equivalent amounts of lysates. (**c**) NP69 and HEK293 cell lines were incubated with cell-free EBV-GFP for 3 hours, and the remaining extracellular viruses were removed by washing with 1×PBS. EBV copy numbers were then measured by TaqMan-qPCR. (**d-g**) After 72 hours, flow cytometric analyses were performed to show the percentage of EBV-GFP-positive cells. Representative images of cells infected with EBV-GFP were recorded for HEK293 cells (**h**). The results are expressed as the mean ± s.e.m. from at least three biological replicates. *p < 0.05, **p < 0.01 (n ≥ 3, a, b, d, e), two-tailed *t*-test. OE, overexpression. Source data

Using lentiviruses, we initially knocked down IFITM1 expression in NP69 and HEK293 cells. The efficiency of IFITM1 knockdown was confirmed using both RT−qPCR and western blotting (Extended Data Fig. 2b,c). Following infection with high-titre virus (multiplicity of infection: 1,000) for 3 h, IFITM1 knockdown cells showed approximately three times more EBV copies than controls (Fig. 1a and Extended Data Fig. 2d). After 72 h of EBV exposure, the green fluorescent protein (GFP)-expressing virus was detectable in infected cells. Approximately 2% of control cells were GFP-positive, which increased to 4% upon IFITM1 knockdown (Fig. 2b,c and Extended Data Fig. 2e–h). Representative images of EBV-infected cells are shown in Extended Data Fig. 2i. In addition, we established IFITM1 overexpression in HK1 cells by transfecting them with IFITM1 lentiviral vectors and control vectors (Extended Data Fig. 3a,b). After EBV exposure for 3 h, EBV copy number decreased by ~60% in IFITM1-overexpressed ECs relative to control cells (Fig. 1d and Extended Data Fig. 3c). After 72 h of EBV exposure, flow cytometry displayed a reduction in the infection rate of over 50% (~3% to 1.5%) upon IFITM1 overexpression (Fig. 1e,f and Extended Data Fig. 3d–g). Representative images of EBV-GFP-infected cells are shown in Extended Data Fig. 3h. These results suggest that IFITM1 knockdown increases the vulnerability of ECs to EBV infection, while IFITM1 overexpression diminishes their susceptibility.

Given that NP69, HEK293 and HK1 represented distinct epithelial cell models, we supplemented our experiments by manipulating IFITM1 levels in NPC-derived and normal ECs. Despite HK1’s inherently low IFITM1, effective IFITM1 knockdown was still achieved (Extended Data Fig. 2b,c), while NP69 and HEK293, initially high in IFITM1, also successfully overexpressed it (Extended Data Fig. 3a,b). Notably, IFITM1 knockdown weakened HK1 cells’ defence against EBV (Extended Data Fig. 2d–i), while its overexpression enhanced the anti-EBV infection ability in NP69 and HEK293 cells (Extended Data Fig. 3c–h). These results further support IFITM1’s critical role in inhibiting EBV infection across various ECs.

Collectively, IFITM1 may serve as an inhibitory factor for EBV infection in ECs.

### Soluble IFITM1 inhibits EBV infecting ECs in vitro and in vivo

**Extended Data Fig. 4:**
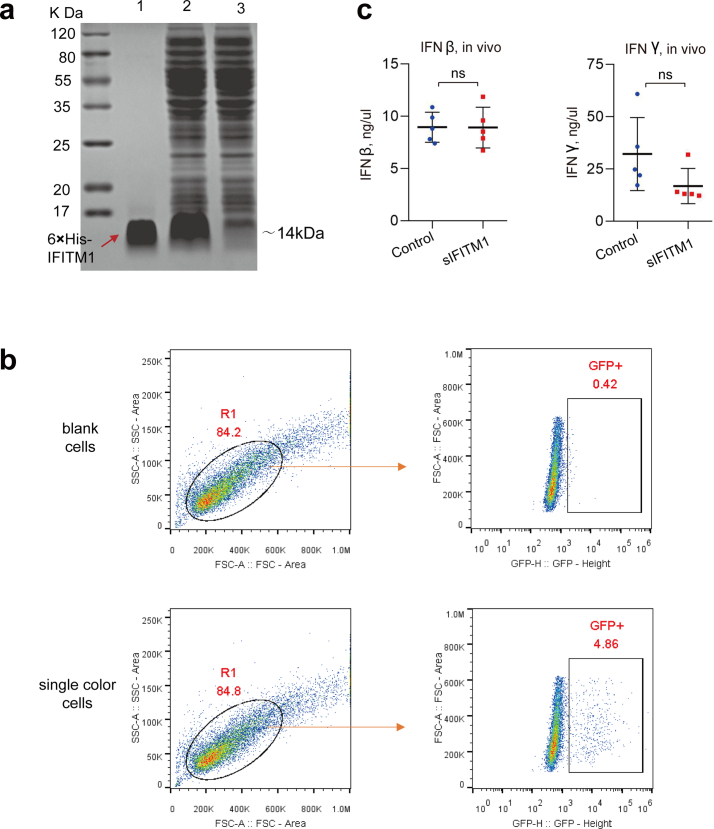
sIFITM1 couldn’t inhibit EBV infection in B cells. (**a**) SDS–PAGE gel shows the purified 6×His-IFITM1 protein (indicated by the red arrow). (**b**) Diagram of FCM analysis process. In this study, all FCM analyses were carried out according to Extended Data Fig. 4b. (**c**) Nude mice were xenografted with HEK293 to form tumor-like cell clusters followed by injection of sIFITM1 and EBV-GFP at set intervals. A control was included in which ddH_2_O was injected at the same interval. After treatment, equal amounts of blood were obtained from Nude mice to detect IFNβ and IFNγ by ELISA. Five mice were investigated for each group (n = 5, ns: no significant difference). Data are presented as mean values ± s.e.m., *p < 0.05, **p < 0.01 (d: n = 8, e: n = 5). Source data

IFITM1 is a small (~14 kDa) membrane protein. To explore its potential to inhibit EBV infection in ECs, we purified soluble 6×His-IFITM1 fusion protein (sIFITM1) (Extended Data Fig. 4a) and treated HEK293 cells with low (1 ng µl^−1^) or high (5 ng µl^−1^) concentration for 2 h before EBV-GFP exposure (Fig. 1g). Infection efficiency was assessed by *Taq*Man−qPCR after 3 h (Fig. 1h) and by flow cytometry at 72 h post infection. Notably, compared with the control group (treated with H_2_O), treatment with sIFITM1, particularly at higher concentration, significantly reduced EBV-GFP-positive cells (Fig. 1i,j), and flow cytometry analysis was carried out according to Extended Data Fig. 4b.

Next, we examined the in vivo effect of sIFITM1 on EBV infection using nude mice xenografted with HEK293 cells subcutaneously (Fig. 1k). Considering the potential immune response impact of sIFITM1 treatment, we detected interferon-β/γ expression in vivo and observed no significant difference between treated and untreated groups (Extended Data Fig. 4c). After the formation of detectable epithelial-cell clusters, sIFITM1 was sequentially injected at the inoculation sites of HEK293 cells, followed by injections of EBV-GFP. After three injections of sIFITM1 and EBV-GFP, mice were euthanized and cell clusters were collected to detect the EBV copy number by *Taq*Man−qPCR. The results showed that sIFITM1 treatment or IFITM1 overexpression diminished EBV infection efficiency by nearly half (Fig. 1l), suggesting an antiviral role for IFITM1 in vivo.

Collectively, these results indicate that sIFITM1 suppressed EBV infection in ECs in vitro and in vivo.

### IFITM1 competes with EBV-gH/gL and gB for binding to EphA2

**Extended Data Fig. 5:**
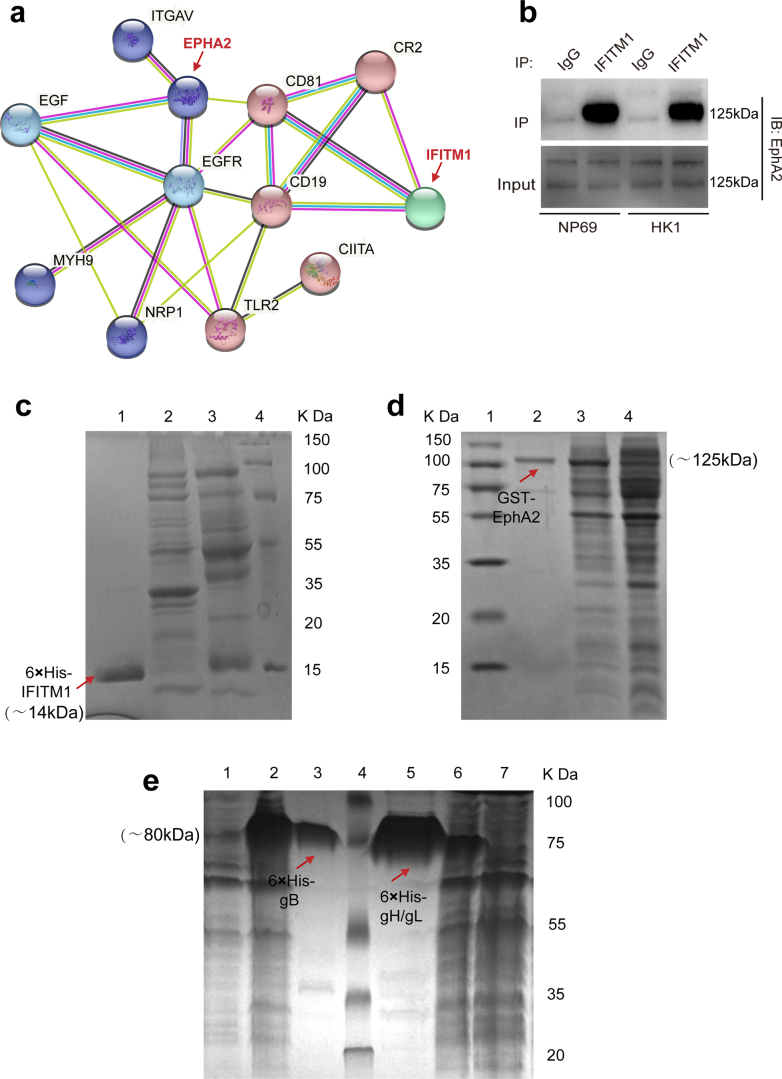
STRING interactome analysis of IFITM1 and proteins involved in EBV infection. (**a**) EphA2, NRP1, MYH9, and ITGAV are EBV infection receptors in epithelial cells (dark blue). EGF and EGFR are also involved in EBV infection in epithelial cells (light blue). CR2, CD81, CD9, TLR2, and CIITA are B cell-related receptors (pink). IFITM1(green) and EphA2 are indicated by the red arrow. (**b**) Co-IP assays showed that endogenous IFITM1 co-immunoprecipitated with EphA2. NP69 and HK1 cell lysates were immunoprecipitated (IP) with IFITM1 antibody, followed by an immunoblotting (IB) assay with EphA2 antibody. IgG-IP samples were included as a control. (**c**) Recombinant His-IFITM1 protein expression detected by Coomassie Brilliant Blue staining. Lane 2: without IPTG induction, lane 3: IPTG induction, lane 1 purified fusion protein. (**d**) Recombinant GST-EphA2 protein expression detected by Coomassie Brilliant Blue staining. Lane 4: without IPTG induction, lane 3: IPTG induction, lane 2 purified fusion protein. (**e**) Recombinant His-gB, and His-gH/gL protein expression detected by Coomassie Brilliant Blue staining. Lane 1 and 7: without IPTG induction, lane 2 and 6: IPTG induction, lane 3 and 5 purified fusion protein. Representative of two independent experiments (**b-e**). Source data

Previous studies have indicated that IFITM1 is localized at the cell plasma membrane^39^ and regulates viral fusion along with cellular proteins^40^. To clarify the mechanism behind IFITM1’s role in inhibiting EBV infection, we utilized the STRING database (http://string-db.org/) to predict protein–protein interactions between IFITM1 and known EBV infection receptors. Our analysis positioned IFITM1 within the network closely associated with EphA2 (Extended Data Fig. 5a, red arrow). The STRING database is a repository of reported or published data. So far, there have been no studies documenting a direct interaction between IFITM1 and EphA2. As such, it was unsurprising that we did not find a direct link between these two proteins, but this predictive outcome did provide us with an initial hint regarding their potential interaction. Given the previously reported close association of EphA2 with EBV infection^18,19^, we selected EphA2 as a focal point to explore the relationship between IFITM1 and EphA2 in the context of EBV infection of ECs.

EphA2 is pivotal for EBV infection, yet its high levels are found in non-susceptible ECs^22^. To determine whether IFITM1 is involved in EphA2-mediated EBV infection, we performed immunofluorescence staining and found co-localization of IFITM1 and EphA2 on the cell surface (Fig. 2a). Co-immunoprecipitation (Co-IP) assays also confirmed their interaction (Extended Data Fig. 5b), suggesting the binding of IFITM1 to the EBV entry receptor on the surface of ECs.

Since EBV-gH/gL and gB are known to bind to EphA2 in ECs, we next examined whether IFITM1 affected this binding using Co-IP experiments. Previous reports indicated that gH/gL and gB showed a slight difference in their binding ability to EphA2 (ref. ^19^), so we used the binding of EphA2 to gH/gL as a reference in Co-IP experiments. We observed that overexpressing IFITM1 reduced EphA2-gH/gL or gB interaction (Fig. 2b, left), while IFITM1 knockdown enhanced it (Fig. 2b, right). Moreover, doubling the copy number of IFITM1 plasmid further decreased the binding affinity of EphA2 to gH/gL (Fig. 2c).

To further validate the direct interaction between IFITM1, EphA2 and gH/gL or gB, we carried out competition binding assays. We purified 6×His-IFITM1, 6×His-gH/gL and 6×His-gB and glutathione S-transferase (GST)-EphA2 (Extended Data Fig. 5c–e). GST-EphA2 was coated on microtitre plates and incubated with purified proteins to analyse affinity constants. Notably, the binding capacity (EC_50_, 50% effective concentration, refers to the concentration for 50% of maximal effect) of IFITM1 to EphA2 (EC_50_, 15.42 nM) was significantly higher than that of gB to EphA2 (EC_50_, 41.67 nM) or gH/gL to EphA2 (EC_50_, 33.52 nM) (Fig. 2d), suggesting that IFITM1 preferentially bound to EphA2 and competed with EBV-gH/gL and gB for binding to EphA2.

To further validate the effect of IFITM1 on the binding ability between gH/gL or gB and EphA2, we conducted an additional affinity constant analysis (enzyme-linked immunosorbent assay, ELISA) incubating gH/gL and gB (with/without IFITM1) on GST-EphA2-coated plates. We observed that the presence of IFITM1 reduced the binding ability between gB and EphA2 (EC_50_, 39.62 nM versus 84.34 nM, Fig. 2e left), and between gH/gL and EphA2 (EC_50_, 32.05 nM versus 75.71 nM, Fig. 2e right). These findings suggest that the interaction between IFITM1 and EphA2 disrupts the binding of EphA2 to gH/gL or gB.

### IFITM1 impairs EphA2-mediated EBV infection

**Extended Data Fig. 6:**
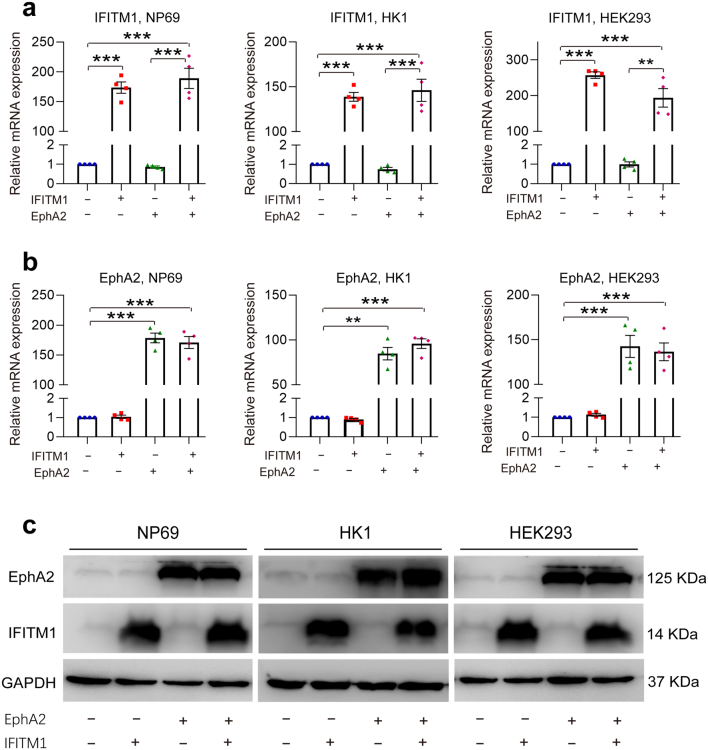
Overexpression of IFITM1 or/and EphA2 in NP69, HK1, and HEK293 cells. (**a–c**) Lentiviruses encoding IFITM1 or EphA2 (referred to by ‘+’) and their corresponding unloaded lentiviruses (referred to by ‘−’) were transfected into NP69, HK1, and HEK293 cells. (**a**,**b**) Total RNA was obtained and RT-qPCR was performed to detect the relative mRNA levels of IFITM1 (**a**) and EphA2 (**b**). Data are presented relative to B_2_M (2^˗ΔCt^). All results are expressed as the mean ± s.e.m. from at least three biological replicates (n ≥ 3, a, b), two-tailed *t*-test. *p < 0.05, **p < 0.01, ***p < 0.001. (**c**) The corresponding IFITM1 protein levels in cells from (**a**) and (**b**). GAPDH was used as a control to indicate equivalent amounts of lysates. The results are from at least three biological replicates. Source data

To further assess the impact of IFITM1–EphA2 on EBV infection, we overexpressed them in NP69, HK1 and HEK293 cells. As expected, the overexpression of IFITM1 and EphA2 had negligible effects on each other’s expression levels (Extended Data Fig. 6a–c), indicating that there was no reciprocal regulation between them.

**Fig. 3:**
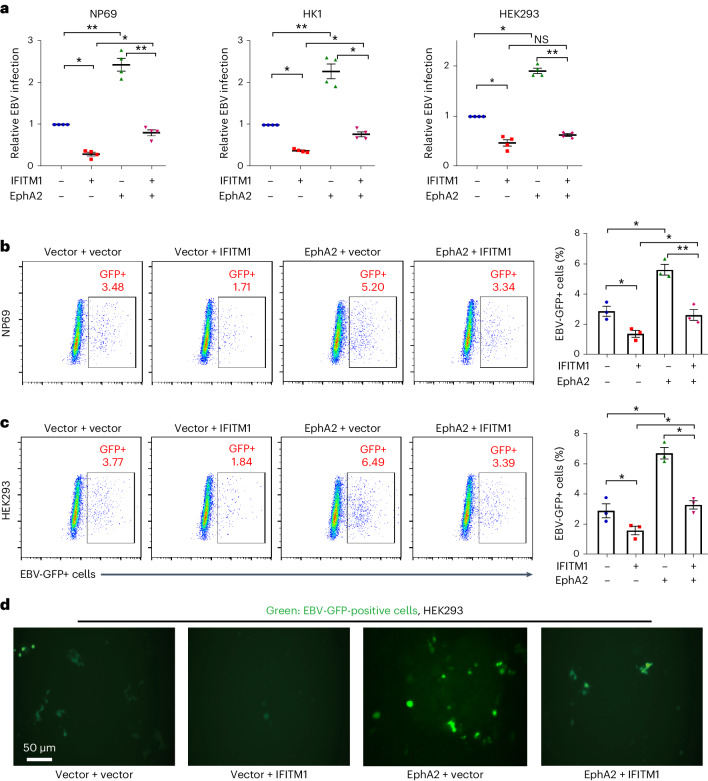
IFITM1 impairs EphA2-mediated EBV infection. **a**, Three cell lines (from Extended Data Fig. 6) were exposed to EBV-GFP for 3 h, and the remaining extracellular viruses were removed by washing with 1×PBS. The copy numbers of EBV were then measured using *Taq*Man−qPCR. **b**,**c**, After 72 h, flow cytometric analyses were performed to determine the percentage of EBV-GFP positive NP69 cells (**b**) and HEK293 cells (**c**). The left image is representative flow cytometric scatterplots, and the right image is the corresponding quantitative analysis of EBV-GFP-positive cells. After 72 h, flow cytometric analyses (left) were performed to determine the percentage (right) of EBV-GFP-positive cells. **d**, Representative images of cells infected with EBV-GFP were recorded for HEK293 cells. All results are expressed as mean ± s.e.m. from at least 3 biological replicates (*n* ≥ 3, **a**–**c**). *P < 0.05, **P < 0.01, NS, not significant; two-tailed *t*-test. Source data

We then exposed cells overexpressing IFITM1 (IFITM1+vector), EphA2 (Vector+EphA2), both (IFITM1+EphA2) and controls (vector+vector) to EBV-GFP for 3 h. After removing remaining extracellular viral particles, we collected cells to detect viral copy number using *Taq*Man−qPCR. Our results showed that IFITM1 overexpression reduced EBV infection by ~70% compared with control cells, whereas EphA2 overexpression doubled the EBV copy number in cells, and their co-overexpression reduced this effect (Fig. 3a). Similarly, after exposing cells to EBV for 72 h, flow cytometric analysis confirmed the partial neutralization of EphA2-mediated EBV infection by co-overexpression of IFITM1 (Fig. 3b,c). Representative images of EBV-infected cells with green fluorescence are presented in Fig. 3d. This demonstrates IFITM1–EphA2 antagonism in the context of EBV infection.

### Two residues on IFITM1 are critical for anti-EBV entry

**Fig. 4:**
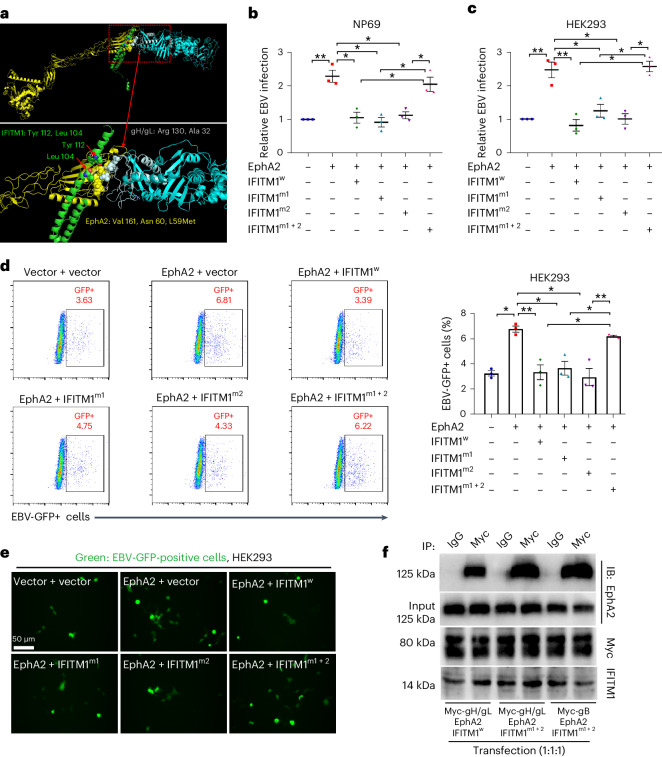
Tyr 112 and Leu 104 on IFITM1 are responsible for the inhibition of EphA2-mediated EBV infection. **a**, Three-dimensional structural model of the interactions between IFITM1 (green), EphA2 (yellow), gH (light blue) and gL (grey). A distance between atoms of the distinct protein backbones of less than 6.0 Å was considered to indicate interfacial amino acids, as indicated by the red arrows and as listed in the figure (IFITM1: Tyr 112, Leu 104; EphA2: Val 161, Asn 60, Met L59; gH/gL: Arg 130, Ala 32). **b**–**e**, Effect on the EBV infection efficiency when the predicted interfacial amino acids on IFITM1 (Tyr 112, Leu 104) were mutated. All assays were performed on cells containing the empty lentivirus or EphA2-overexpressed cells (NP69 and HEK293) with a single-site mutation (IFITM1^m1^ or IFITM1^m2^) or dual mutations (IFITM1^m1+2^) of IFITM1, and control cells (IFITM1^w^). EBV copy numbers were measured by *Taq*Man−qPCR when cells were incubated with cell-free EBV-GFP for 3 h (**b**, NP69; **c**, HEK293). **d**,**e**, Quantitative and qualitative analysis of EBV-GFP-positive cells after 72 h of EBV exposure in transformed HEK293 cells. **d**, Representative flow cytometric scatterplots and the corresponding quantitative analysis of EBV-GFP-positive cells. **e**, Representative images of cells infected with EBV-GFP. **f**, Co-IP assays showed that IFITM1 influences the binding of EphA2 to gH/gL or gB. A total of 3 combinations of plasmids were designed and 3 different plasmids were transfected into each group at a ratio of 1:1:1. Briefly, the plasmids were transfected into HEK293 cells for 8 h and the cells were collected at 48 h after transfection. Cell lysates were immunoprecipitated with anti-Myc antibody, followed by immunoblotting analysis with anti-EphA2, Myc and IFITM1 antibodies. All results are expressed as mean ± s.e.m. of 3 biological replicates (*n* = 3, **b**–**d**). *P < 0.05, **P < 0.01, two-tailed *t*-test. Source data

**Extended Data Fig. 7:**
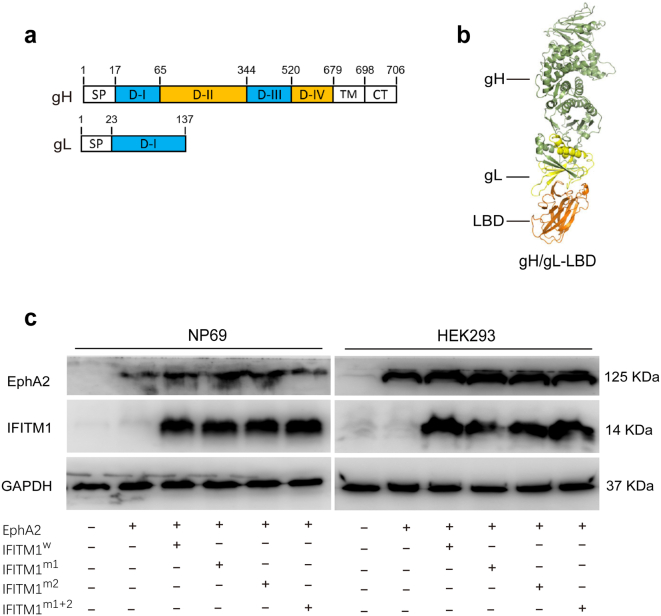
Site-directed mutation of 112Tyr and 104Leu on IFITM1. (**a**) Schematic representation of the domain structure of EBV gH/gL; CT: C-terminal cytoplasmic tail domain, SP: signal peptide. (**b**) Cartoon representation of the structure of the EBV gH/gL-ligand-binding domain (LBD)^41^. EBV gH is indicated in green, gL is indicated in yellow, and the LBD of EphA2 is indicated in orange. (**c**) EphA2-overexpressing cells (NP69; HEK293) were transfected with plasmids corresponding to wild-type IFITM1 (IFITM1^w^), 112Tyr single-site mutation (IFITM1^m1^), 104Leu single-site mutation (IFITM1^m2^), or dual mutations (IFITM1^m1+2^). Experiments were implemented 72 hours after transfection. The cell lysates were harvested to test the IFITM1 level. GAPDH was used as a control to indicate equivalent amounts of lysates. The results are from at least three biological replicates. Source data

Previous research indicated that EBV’s fusion with host ECs was initiated by the ligand-binding domain (LBD, amino acids 20–206) of EphA2 binding to EBV gH-DI domain or gL (Extended Data Fig. 7a,b)^41^. To better understand the competitive mechanism, we performed three-dimensional (3D) structure prediction of the full-length monomers of IFITM1, EphA2 and gH/gL using I-TASSER (https://zhanglab.ccmb.med.umich.edu/I-TASSER/) and SWISS-model (https://swissmodel.expasy.org/). We calculated and selected a ternary complex model using clustering score and aligned it with previously reported models. Notably, our results showed a ‘clip-like’ interaction between IFITM1 and EphA2’s extracellular domain (Fig. 4a). Two key residues (Tyr 121 and Leu 104) on IFITM1 shared binding sites with both EphA2-LBD (Val 161, Asn 60 and Met 59) and EBV gH/gL (Arg 130 and Ala 32) (Fig. 4a bottom). This suggests that IFITM1 may occupy these two binding sites on EphA2, which EBV glycoproteins also bind.

Next, we investigated whether these two residues on IFITM1 were critical for inhibiting EBV infection. Site-directed mutagenesis was performed on the Tyr 121 or/and Leu 104 residues of IFITM1 in NP69 and HEK293 cells, producing IFITM1^m1^ (Tyr 121), IFITM1^m2^ (Leu 104) and IFITM1^m1+2^ (Tyr 121+Leu 104) (Extended Data Fig. 7c). Subsequently, EphA2 was overexpressed in these cells followed by exposure to EBV. After 3 h, EBV entering the cells was detected using *Taq*Man−qPCR. The results showed that wild-type IFITM1 (IFITM1^w^) markedly inhibited EphA2-mediated EBV infection, while single mutations of IFITM1 (IFITM1^m1^ or IFITM1^m2^) slightly impaired this inhibitory effect, and dual mutations of IFITM1 (IFITM1^m1+2^) completely abolished the inhibition of EBV infection (Fig. 4b,c, NP69 and HEK293). After 72 h, flow cytometry confirmed these findings (Fig. 4d). Representative fluorescence images are presented in Fig. 4e. These results highlight the critical role of Tyr 121 and Leu 104 in IFITM1’s anti-EBV function.

To further validate whether the observed interference was a consequence of the mutations affecting the binding between EphA2 and gH/gL or gB, we executed Co-IP using IFITM1^w^ and IFITM1^m1+2^ with endogenous EphA2 and exogenous gH/gL or gB. The results demonstrated that the mutation of these two residues, Tyr 121 and Leu 104, on IFITM1 resulted in an increased binding affinity between EphA2 and either gH/gL or gB (Fig. 4f).

Collectively, these findings suggest that Tyr 121 and Leu 104 residues on IFITM1 are critical for its inhibitory effect on EphA2-mediated EBV infection by influencing the binding between EphA2 and gH/gL or gB.

### YTHDF3 negatively regulates the level and function of IFITM1

**Fig. 5:**
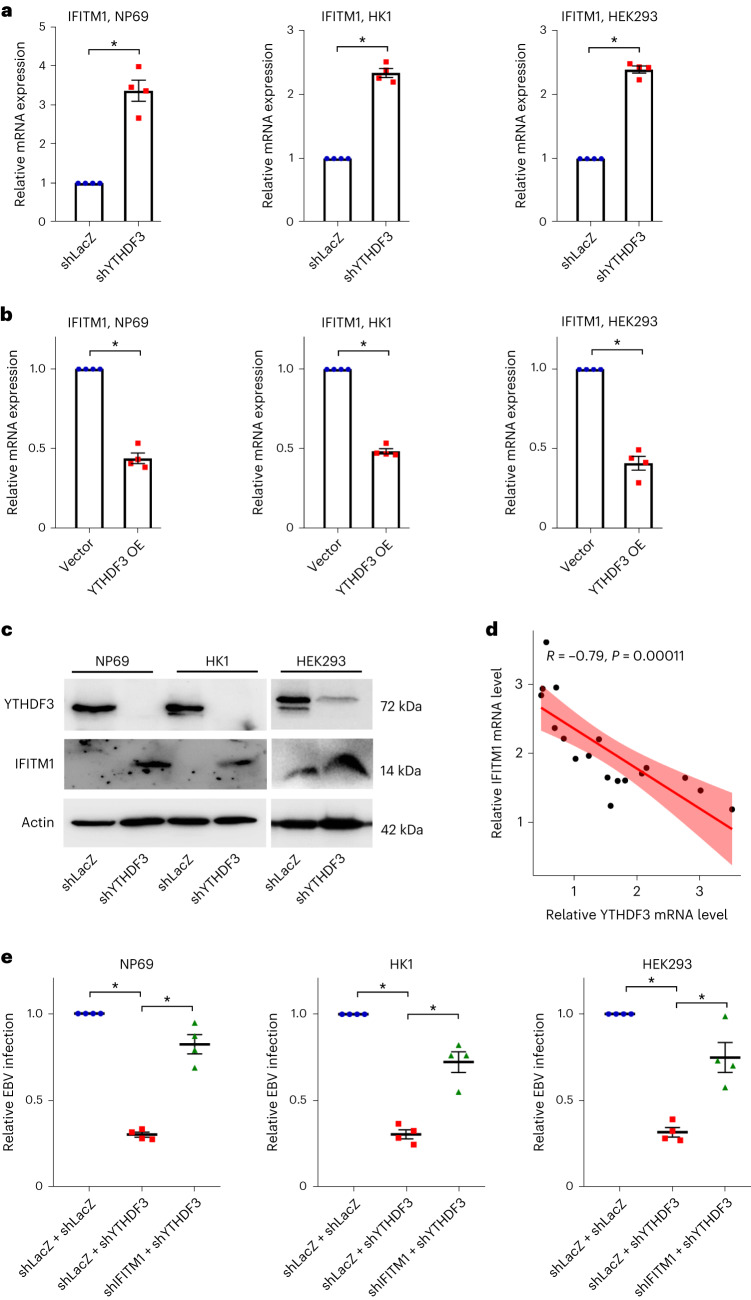
The expression and function of IFITM1 were negatively regulated by YTHDF3. **a**,**b**, IFITM1 expression levels were tested by RT−qPCR in cells from Extended Data Fig. 8b (**a**) and Extended Data Fig. 8c (**b**). Data are presented as mean ± s.e.m., *n* = 3 independent experiments, *P < 0.05, two-tailed *t*-test. **c**, Western blot showing the relative protein levels of YTHDF3 and IFITM1 in NP69, HK1 and HEK293 cells. The Actin protein was used as a control to indicate equivalent amounts of lysates. Representative of 2 independent experiments. **d**, Correlation analysis of the relative YTHDF3 and IFITM1 mRNA expression levels in 9 NPC and 9 NP samples. Data are presented as mean ± s.e.m., *n* = 18, *r* = −0.79, *P* = 0.00011, two-tailed *t*-test (**d**). **e**, *Taq*Man−qPCR showing the effects of YTHDF3 and IFITM1 on EBV infection. Lentiviruses encoding shYTHDF3 or shIFITM1 and their corresponding negative control lentiviruses (shLacZ + shLacZ) were transfected into NP69, HK1 and HEK293 cells. Cells were exposed to EBV-GFP for 3 h, EBV copy numbers were measured by *Taq*Man−qPCR and results from control groups were taken as 100%. Data are presented as mean ± s.e.m., *n* = 4 independent experiments, *P < 0.05, two-tailed *t*-test. Source data

**Extended Data Fig. 8:**
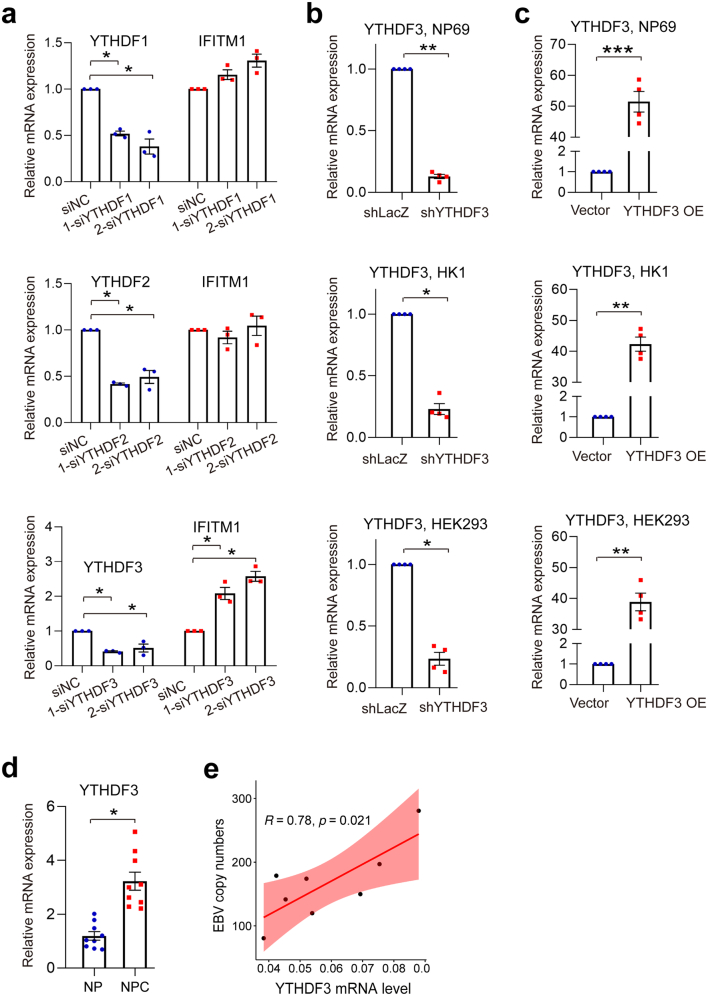
IFITM1 expression is negatively regulated by YTHDF3. (**a**) RT-qPCR shows the relative IFITM1 mRNA levels in siYTHDF1, siYTHDF2, and siYTHDF3 HEK293 cells. (**b**) Relative YTHDF3 mRNA levels in NP69, HK1, and HEK293 cells was checked by RT-qPCR after infecting with lentiviruses encoding shYTHDF3 or shLacZ. (**c**) Relative YTHDF3 mRNA levels in NP69, HK1, and HEK293 cells was checked by RT-qPCR after infecting with the control (Vector) or YTHDF3-overexpressing (YTHDF3 OE) lentiviruses. Data are presented relative to B_2_M (2^˗ΔCt^). Mean ± s.e.m., n ≥ 3 independent experiments, *p < 0.05, **p < 0.01, ***p < 0.001, two-tailed *t*-test (**a-c**). (**d**) RT-qPCR of YTHDF3 mRNA expression in nine nasopharyngeal carcinoma (NPC) and nine nasopharyngeal (NP) samples. Total RNA from each tissue was assayed by RT-qPCR and B_2_M was used as a reference gene. Data are presented as mean values ± s.e.m., n = 18, two-tailed *t*-test. (**e**) Correlation analysis of the relative YTHDF3 mRNA expression level and the EBV copy number (horizontal axis: YTHDF3 relative mRNA level; vertical axis: relative EBV copy number). Data are presented as mean values ± s.e.m., n = 8, r = –0.78, *p* = 0.021 (e). Source data

To explore the regulatory role of m^6^A on IFITM1 expression, we performed small interfering RNA (siRNA) knockdown of m^6^A readers YTHDF1, YTHDF2 and YTHDF3 in HEK293 cells (Extended Data Fig. 8a). Highest IFITM1 expression was observed following YTHDF3 knockdown, suggesting the key role of YTHDF3 in regulating IFITM1 expression. To substantiate this, we constructed YTHDF3 knockdown and overexpressed cells using lentivirus (Extended Data Fig. 8b,c). The results revealed that YTHDF3 downregulation increased IFITM1 by nearly threefold (Fig. 5a), while YTHDF3 overexpression reduced IFITM1 by ~50% (Fig. 5b). Western blotting corroborated this negative correlation between IFITM1 and YTHDF3 (Fig. 5c), which was further supported by RT−qPCR analysis of clinical samples (Fig. 5d, *R* = −0.79, *P* = 0.00011), suggesting that YTHDF3 may exert a negative regulatory effect on IFITM1 expression.

To investigate the interaction between YTHDF3 and IFITM1 in the context of EBV infection, we measured EBV infection efficiency in shLacZ and shYTHDF3 cells after a 3 h EBV-GFP exposure. Notably, results showed that YTHDF3 knockdown reduced EBV copy number, which was partially restored by IFITM1 co-silencing (Fig. 5e). Clinical data exhibited aberrantly elevated YTHDF3 in NPC tissues with high EBV copy numbers, with YTHDF3 levels and EBV copy numbers correlating positively (Extended Data Fig. 8d,e). These data suggest that YTHDF3 negatively regulates both the expression and anti-EBV function of IFITM1 in ECs.

### YTHDF3 regulates m^6^A-modified IFITM1 degradation via DDX5

**Fig. 6:**
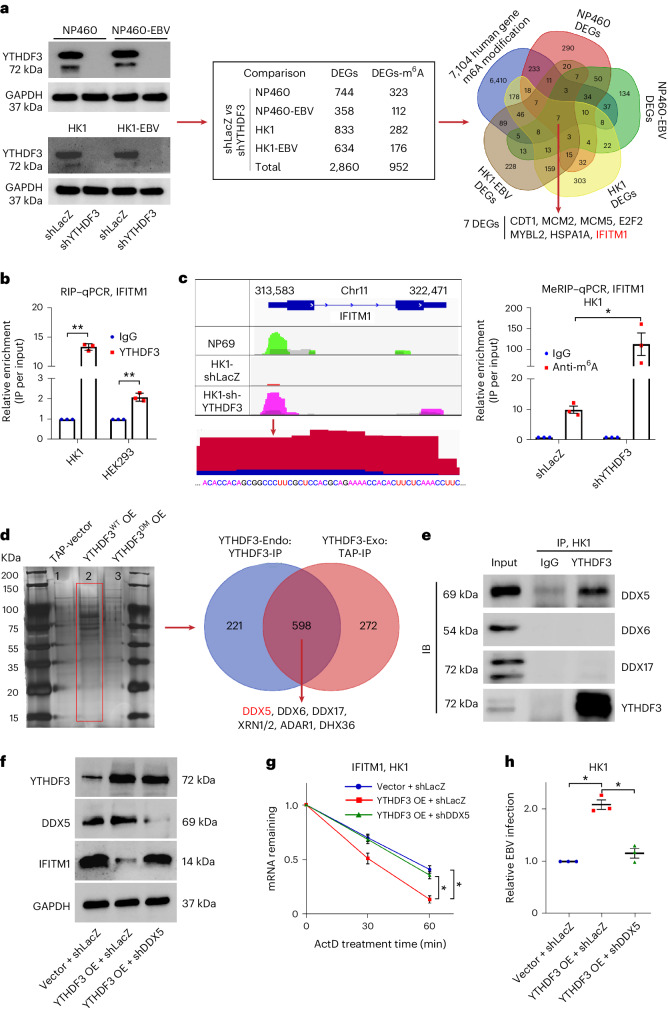
YTHDF3 recognizes m^6^A modification sites and interacts with degradation-related proteins participating in the regulation of IFITM1. **a**, Left: YTHDF3 knockdown was performed in NP460, NP460-EBV, HK1 and HK1-EBV cells; representative of 2 independent experiments. Middle: DEGs were identified through RNA-seq. Right: Venn diagram showing the overlap between DEGs and m^6^A modification sites of 7,104 human genes reported in ref. ^42^. Seven DEGs including *IFITM1* were screened out. **b**, Fold enrichment of IFITM1 as determined by RIP−qPCR. **c**, Left: MeRIP-sequencing, grey region shows m^6^A enrichment based on the input RNA. m^6^A motif sequences corresponding to the immunoprecipitated enriched region are indicated by different colours in different cells (NP69, green; HK1, red; HK1-shYTHDF3, magenta; input, grey). The RNA sequence at the bottom is the predicted m^6^A site binding with YTHDF3. Right: MeRIP−qPCR, fold enrichment of IFITM1 as determined by MeRIP−qPCR. **d**, Left: silver stain of the eluted protein from tandem affinity purification and mass spectrometry. Lines 1, 2 and 3 show the purified proteins from HK1-TAP, HK1-TAP-YTHDF3^WT^ and HK1-TAP-YTHDF3^DM^, respectively. Right: Venn diagram of the exogenous YTHDF3-binding proteins (HK1-YTHDF3-Exo-TAP: from purified proteins in HK1-TAP-YTHDF3^WT^) and endogenous YTHDF3-binding proteins (HK1-YTHDF3-Endo-TAP: from endogenous YTHDF3-IP). The red rectangle serves to differentiate the two bands on the gel more clearly. **e**, Co-IP assays validated that endogenous YTHDF3 co-immunoprecipitated with DDX5, DDX6 and DDX17. HK1 cell lysates were immunoprecipitated (IP) with YTHDF3 antibody, followed by an immunoblotting (IB) assay with the corresponding DDX antibodies. IgG-IP samples were included as a control. **f**, Lentiviruses encoding YTHDF3 overexpression or DDX5 knockdown and their corresponding negative control lentiviruses (vector + shLacZ) were transfected into HK1 cells, and immunoblotting assays were performed using anti-YTHDF3, DDX5 and IFITM1. Representative of 3 independent experiments (**d**–**f**). **g**, IFITM1 remaining, detected by RT−qPCR after treating with transcriptional inhibitor ActD. **h**, Cells from **f** were exposed to EBV-GFP for 3 h, EBV copy numbers were measured by *Taq*Man−qPCR and results from control groups were taken as 100%. Mean ± s.e.m., *n* = 3 independent experiments, *P < 0.05, **P < 0.01, two-tailed *t*-test (**b**,**c**,**g**,**h**). Source data

YTHDF3 is an m^6^A reader that exerts its regulatory roles by binding to the m^6^A site on RNA. To determine whether IFITM1 mRNA has m^6^A sites recognized by YTHDF3, we silenced YTHDF3 in four NPC lines (NP460, NP460-EBV, HK1, HK1-EBV) (Fig. 6a left). RNA-seq identified differentially expressed genes (DEGs) post silencing, which were cross-referenced with 7,104 human gene m^6^A sites from ref. ^42^ (Fig. 6a right)^42^. Of 2,860 DEGs, 952 possessed YTHDF3-associated m^6^A sites (Fig. 6a middle). Further Venn diagram analysis revealed that seven genes, including *IFITM1*, were consistently regulated by YTHDF3 across all four cell lines (Fig. 6a right), suggesting direct regulation of IFITM1 by YTHDF3 through m^6^A.

**Extended Data Fig. 9:**
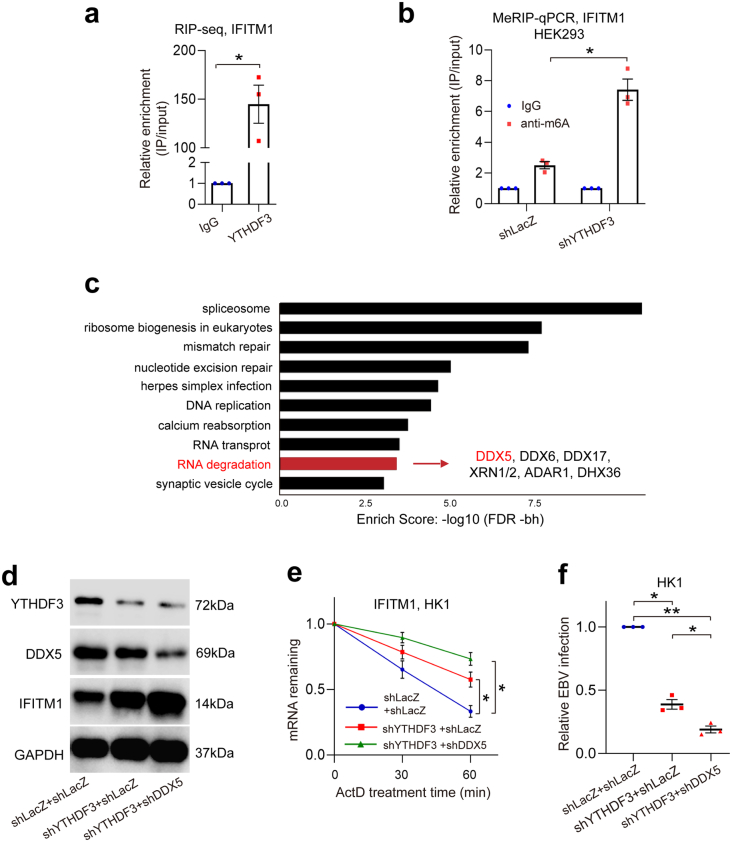
IFITM1 expression is negatively regulated by YTHDF3 via RNA degradation. (**a**) Fold enrichment of IFITM1 was analyzed by RIP-seq in shLacZ or shYTHDF3-HK1 cells. (**b**) Fold enrichment of IFITM1 was determined by MeRIP-qPCR in shLacZ or shYTHDF3-HEK293 cells and data was presented relative to B_2_M (2^˗ΔCt^). (**c**) KEGG pathway analysis of the 598 proteins from Fig. 6d. Proteins in the DDX family related to RNA degradation were detected. (**d**) Lentiviruses encoding shYTHDF3 or shDDX5 and their corresponding negative control lentiviruses (shLacZ + shLacZ) were transfected into HK1 cells, and immunoblotting assay were performed with anti-YTHDF3, DDX5, and IFITM1. (**e**) Cells from Extended Data Fig. 9d were treated with transcriptional inhibitor ActD, followed by IFITM1 detection using RT-qPCR. (**f**) Cells from Extended Data Fig. 9d were exposed to EBV-GFP for 3 hours and the remaining extracellular viruses were removed by washing with 1×PBS. EBV copy numbers were then measured by TaqMan-qPCR and results from control groups were taken as 100%. Data are presented as mean values ± s.e.m., n = 3 independent experiments, *p < 0.05, **p < 0.01, two-tailed *t*-test (a, b, e, f). Source data

To validate the interaction between IFITM1-mRNA and YTHDF3, we performed RNA immunoprecipitation with deep sequencing (RIP-seq) and RIP−qPCR on HK1 and HEK293 cells, showing that IFITM1-mRNA was specifically bound by YTHDF3 (Extended Data Fig. 9a and Fig. 6b). Methylated RNA immunoprecipitation sequencing (MeRIP-seq) and MeRIP−qPCR on shLacZ-HK1 and shYTHDF3-HK1 cells verified the m^6^A modification linked to YTHDF3. The results revealed that m^6^A sites were present in IFITM1 mRNA in ECs, with increased enrichment in shYTHDF3-HK1 compared with controls (Fig. 6c and Extended Data Fig. 9b). In addition, m^6^A sites binding with YTHDF3 (the immunoprecipitated enriched region) were predicted (Fig. 6c, red arrow). These results indicate a direct YTHDF3–IFITM1 mRNA interaction.

To delve deeper into the mechanism by which YTHDF3 epigenetically regulates IFITM1, we established stable overexpression of exogenous tandem affinity purification (TAP)-tags (TAP-vector, TAP-YTHDF3^WT^, TAP-YTHDF3^DM (Double-site mutants)^) in HK1 cells. Subsequently, external TAP pull-down/mass spectrometry and endogenous YTHDF3 co-precipitation/mass spectrometry (YTHDF3-IP/MS) were performed on the lysates of these three cell lines (Fig. 6d, left). The Venn diagram analysis of these two groups of MS data identified 598 proteins binding to both endogenous and external YTHDF3 (Fig. 6d right). Kyoto Encyclopedia of Genes and Genomes (KEGG) clustering of these proteins revealed several RNA degradation-related factors, including DEAD-box RNA helicases (DDX) and 5′-3′-exoribonuclease (XRN) family members (Extended Data Fig. 9c), with DDX5, DDX6 and DDX17 as high-confidence YTHDF3 partners (false discovery rate = 0.03237, *P* = 0.00173). Co-IP assays confirmed a strong YTHDF3–DDX5 interaction (Fig. 6e).

To explore the relationship between YTHDF3 and DDX5, we conducted overexpression (OE) and knockdown (KD) experiments. Notably, the effects of YTHDF3 OE were counteracted by DDX5 KD (Fig. 6f). Western blot analysis revealed that combined knockdown of DDX5 and YTHDF3 led to increased IFITM1 expression compared with YTHDF3 KD alone (Extended Data Fig. 9d). Subsequently, treatment with transcriptional inhibitor ActD followed by RT−qPCR showed that YTHDF3 OE accelerated IFITM1 mRNA decay, which was mitigated by DDX5 KD (Fig. 6g). Furthermore, YTHDF3 KD stabilized IFITM1 mRNA, while simultaneous DDX5 KD further prolonged the half-life of IFITM1 mRNA (Extended Data Fig. 9e), These findings suggest DDX5’s role in YTHDF3-mediated degradation of IFITM1.

To further verify the impact of the YTHDF3–DDX5–IFITM1 regulatory network on EBV infection, we conducted an efficiency test for EBV infection in a series of HK1 cells. The results showed that high YTHDF3 expression enhanced EBV infection, while simultaneous depletion of DDX5 reversed this process (Fig. 6h). Dual depletion of YTHDF3 and DDX5 significantly lowered infection efficiency beyond single YTHDF3 depletion (Extended Data Fig. 9f).

Collectively, these results indicate that YTHDF3 regulates m^6^A-modified IFITM1 degradation via DDX5, impacting EBV infection efficiency.

## Discussion

**Extended Data Fig. 10:**
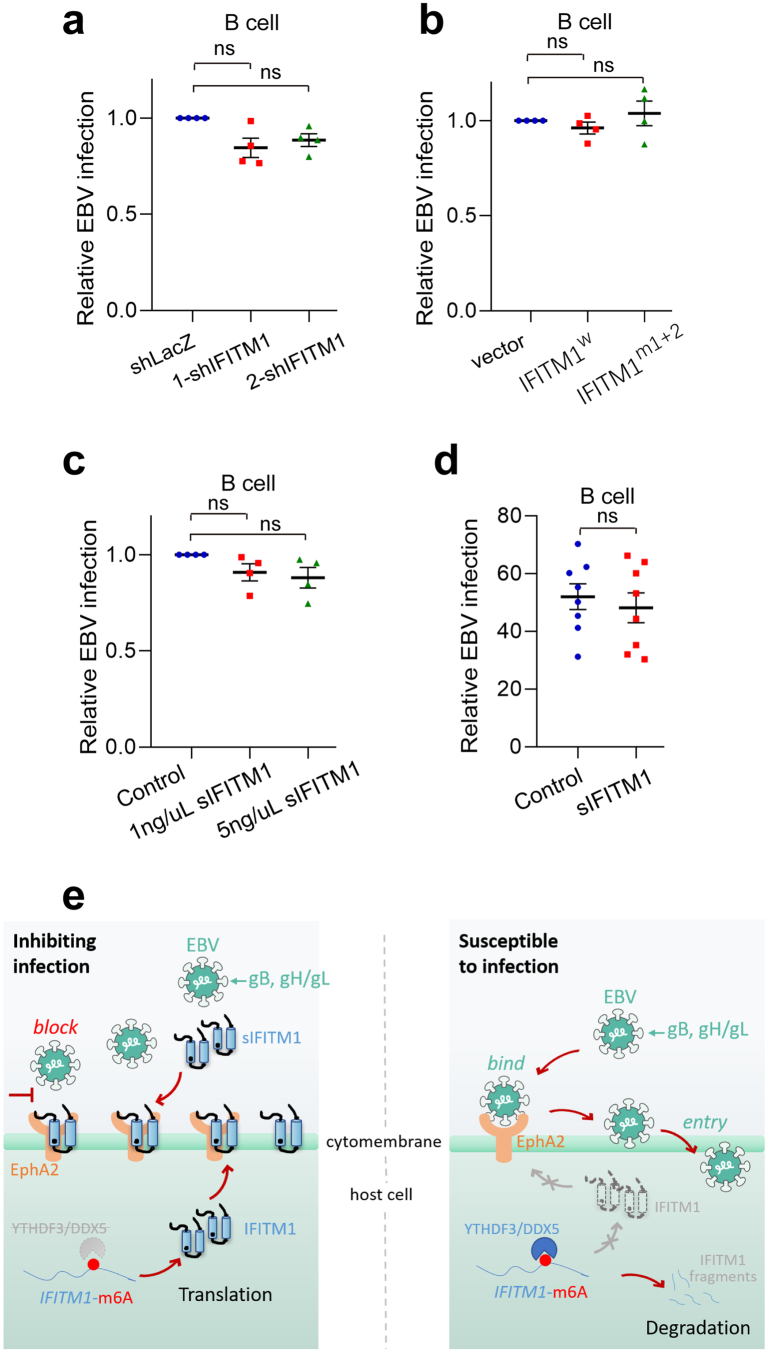
IFITM1 does not affect Epstein-Barr virus infection in B cells. (**a**) Knockdown of IFITM1 in B cells by infection with lentiviruses encoding shIFITM1 (1-shIFITM1 and 2-shIFITM1), then cells were incubated with cell-free EBV-GFP for 3 hours and remaining extracellular viruses were removed by washing with 1×PBS. EBV copy numbers were then measured by TaqMan-qPCR. (**b**) B cells were infection with lentiviruses encoding wild-type IFITM1 (IFITM1^w^), or dual mutations (IFITM1^m1+2^), then cells were incubated with cell-free EBV-GFP for 3 hours and remaining extracellular viruses were removed by washing with 1×PBS. EBV copy numbers were then measured by TaqMan-qPCR. (**c**) Analysis of the anti-EBV effects of sIFITM1 on B cells, exposure of B cells to EBV-GFP for 3 hours, with sIFITM1 being added 2 hours in advance at different concentrations (0, 1, and 5 ng/µL), EBV copy numbers were then measured by TaqMan-qPCR. Data are presented as mean values ± s.e.m., n = 4 independent experiments, *p < 0.05, **p < 0.01 **(a-c**). (**d**) According to the method in Fig. 1k and l, anti-EBV effects of sIFITM1 was tested on B cells in *vivo*. (**e**) A schematic diagram showing two models of EBV infection: an insusceptibility model (left), in which IFITM1 inhibits EBV entry by competing with EBV gH/gL and gB for binding to EphA2; and a susceptibility model (right), in which YTHDF3 recognizes m^6^A modification sites on IFITM1 and interacts with RNA degradation-related proteins DDX5, then leads to the degradation of IFITM1. The loss of IFITM1 would result in exposure of EphA2, which may aid EBV entry. Source data

IFITMs have been extensively studied in RNA viruses, yet their roles in DNA viruses like EBV remain less clear. Our study identifies IFITM1 as a protective factor against EBV infection in ECs. Analysis of clinical samples and EBV+/− cell lines showed an inverse correlation between IFITM1 level and EBV infection. In ‘cell-free’ models simulating direct viral contact with cells, we manipulated IFITM1 expression and assessed EBV entry. Our data revealed that IFITM1 had an adverse impact on EBV infection, which is not consistent with the conclusion in ref. ^32^ that IFITM1 facilitated EBV infection in B (BJAB) and endothelial (HMVEC-d) cells. This discrepancy could be due to: (1) Differences in cell lines used. Our results, along with other studies, suggest that EBV infection receptors vary between B cells and ECs, so it is plausible that IFITM1 may have different functions in BJAB cells and ECs. HMVEC-d, an endothelial cell line typically used as a Kaposi’s sarcoma-associated herpesvirus infection model, is not representative of EBV infection. (2) Differences in infection mechanisms. Reference ^32^ suggested a role for soluble heparin in EBV infection of BJAB cells through gp150, without further elucidating the mechanism. In contrast, our study focused on the interaction between EphA2 and EBV-gH/gL or gB. As a result, the results from both studies on IFITM1’s involvement in EBV infection are independent and not inherently conflicting. In addition, our research presented evidence that neither knockdown or mutation of IFITM1 nor extracellular treatment with sIFITM1 affect EBV infection efficiency in B cells (Extended Data Fig. 10a–d), reinforcing the notion that IFITM1’s role in EBV infection is specific to epithelial cell type.

Recent research has identified EphA2 as an entry receptor for EBV in ECs^18,19^, yet some cells remain insusceptible to EBV even with high EphA2 expression^22^. Viral entry often involves multiple molecules. For example, EphA2 and EGFR collaborate in hepatitis C virus entry^43^ and interact in ECs^44^. Our research confirms that EphA2 mediates EBV entry and notably uncovers the opposing roles of IFITM1 and EphA2 in EBV infection, validating their direct interaction through immunofluorescence (IF), co-IP and ELISA. Phenotypically, IFITM1 neutralized the effects of EphA2-induced EBV infection. Mechanistically, IFITM1 inhibited the binding of EphA2 to viral proteins gH/gL or gB. Notably, we have pinpointed the critical amino acid residues on IFITM1, with residues Tyr 121 and Leu 104 affecting this interaction and reducing EBV infection.

The EphA2-antagonist-like function of IFITM1 against EBV infection was also substantiated in vivo using sIFITM1, suggesting its potential for drug development. Nasal sprays or small-molecule inhibitors have recently been proposed to prevent coronavirus transmission and reduce viral load^45,46^. Given EBV’s transmissibility through close contact, developing barrier agents or entry inhibitors could effectively curb its spread and associated diseases. Thus, IFITM1-based formulations present a promising research direction.

In this study, we also discovered m^6^A modifications on IFITM1 mRNA and identified YTHDF3’s role in recognizing these sites, thereby regulating IFITM1 expression through DDX5. DDX proteins are instrumental in epigenetic regulation via m^6^A and some studies have linked DDX46 and DDX17 with YTHDF proteins, affecting antiviral mRNA nucleo-retention and EBV replication^47,48^. Our findings revealed that YTHDF3, by combining the degradation factor DDX5, suppressed IFITM1 expression and influenced EphA2-mediated EBV infection. This study sheds light on the role of m^6^A modifications in interferon-stimulated-gene-mediated EBV infection, although alternative regulatory pathways or proteins may exist.

Despite our best efforts, we acknowledge that future research will need to address two significant aspects. First, even though IFITM1 has been shown to provide protection before viral entry in ECs, its regulation following EBV infection requires further investigation. Preliminary data from one of our ongoing studies initially suggest that Epstein–Barr nuclear antigen 1 (EBNA1) may epigenetically repress IFITM1 expression, potentially further enhancing EBV infection. This is an area warranting deeper exploration in the future. Second, while it is widely accepted that cell-free transmission is the principal mode of EBV infection and that lytic replication represents the default programme for EBV infection in oropharyngeal ECs^49,50^, there is, to our knowledge, no direct evidence so far further suggesting that EBV spreads through cell–cell fusion. Nonetheless, the broader viral contexts^18,51^ suggest a possible model involving cell–cell fusion facilitated by EBV glycoproteins and EphA2, which might facilitate the merging of two or more cells and aid in viral dissemination. We speculate that IFITM1 might potentially play a role in this process as well. This presents an exciting direction for continued research.

In summary (Extended Data Fig. 10e), our research indicates that IFITM1 acts as a ‘guardian’ against EBV in ECs. Epigenetically controlled by YTHDF3 and DDX5, IFITM1 effectively thwarts EphA2-mediated EBV entry into ECs, particularly via critical residues Tyr 112 and Leu 104. These insights refine our understanding of EBV entry into ECs and may guide potential preventive and therapeutic strategies against EBV infection and related disorders.

## Methods

### Ethics statement

Our research, which included the use of human tissues, adhered to all relevant ethics regulations approved by the Medical Ethics Committee of Southern Medical University (SMU). All clinical samples were obtained with informed consent from the patients. Experimental animals were maintained in alignment with the guidelines recommended in the National Institutes of Health’s Guide for the Care and Use of Laboratory Animals. The protocols were approved by the Ethics Committee of Shenzhen Hospital of SMU on Laboratory Animal Care (No. 2022-0028) .

### Cell lines

The normal nasopharyngeal epithelial cell line NP69 (SV40-immortalized) was grown in complete serum-free medium consisting of basal defined keratinocyte serum-free medium (D-KSFM) supplemented with D-KSFM growth supplement (Gibco). The normal nasopharyngeal epithelial cell line NP460 (hTert-immortalized) and EBV-positive NP460-EBV were maintained in a 1:1 mixture of D-KSFM complete medium and EpiLife medium (containing EpiLife defined growth supplement, EDGS) (Gibco). For additional details about the cell lines specified above, please refer to previous studies^52–54^. The NPC cell line HK1, EBV-positive NPC cell lines HK1-EBV and C666-1, and Akata cells carrying recombinant EBV were cultured in RPMI 1640 medium containing 10% fetal bovine serum (Hyclone). GFP-positive cell lines Akata-EBV were added with G418 (700 ng ml^−1^; Gibco) when necessary. The cell lines specified above were kindly provided by Prof. Sai-Wah Tsao’s group (The University of Hong Kong, Pokfulam, Hong Kong SAR, China). AGS and HEK293 cells were maintained in our laboratory. Daudi cells were purchased from FuHeng Cell Center and cultured in RPMI 1640 containing 10% fetal bovine serum (Hyclone). All cells were cultured with 1% penicillin/streptomycin at 37 °C and 5% CO_2_. We did not use any cross-contaminated cell lines according to the list of known misidentified cell lines maintained by the International Cell Line Authentication Committee (https://iclac.org/databases/cross-contaminations/). All cells underwent mycoplasma testing (Myco-Blue mycoplasma detector, Vazyme) and short tandem repeat analyses.

### Patient samples

The patient-derived tumour and non-tumour samples were provided by Nanfang Hospital of SMU. The sample set comprised 12 NPC tissues clinically diagnosed by histopathological examination (TNM stage III, *n* = 8; TNM stage IV, *n* = 4; male, *n* = 7; female, *n* = 5) and 12 NP tissues with chronic inflammation. The use of these human tissues was approved by the Medical Ethics Committee of SMU. Although no statistical methods were employed to predetermine sample sizes, our sample sizes are in line with those reported in a previous study^55^.

### RNA-seq

RNA-seq was entrusted to Novogene. First, total RNA was extracted from NP69, NP460, NP460-EBV, HK1, HK1-EBV, C666-1, AGS and Daudi cells, and three biological replicates were performed. RNA purity, concentration and quality were measured by a NanoPhotometer spectrophotometer (IMPLEN) and the Bioanalyzer 2100 system (Agilent). Then, RNA libraries were generated using the NEBNext Ultra RNA library prep kit (NEB) following manufacturer recommendations, and at least 1 µg of input total RNA was used for each sample. Library quality was assessed on the Bioanalyzer 2100 system (Agilent). The libraries were sequenced on an Illumina Hiseq platform, yielding 125 bp/150 bp paired-end reads with a data volume of 6 G for each sample. Before analysis, clean data were obtained by filtering out adapter or ploy-N and low-quality reads. Paired-end cleaned reads were aligned with the reference genome using Hisat2 v.2.0.5. Gene expression levels were calculated in FPKM (expected number of Fragments Per Kilobase of transcript sequence per Million base pairs sequenced).

### Lentivirus, plasmids and transfection

Short hairpin RNA (shRNA) constructs (1-shIFITM1 and 2-shIFITM1) and the overexpression construct of IFITM1 were as described previously^56^. For the EBV infection assay, cDNAs of IFITM1 and EphA2 were integrated into the pMSCV-puro vector and MSCV-ires-GFP vector, respectively; for co-IP assays, cDNAs of EphA2, gB or gH/gL were integrated into the pCDNA6-Myc vector, and gH/gL-Flag and gB-Flag were integrated into the pCEP vector; for purification assays of GST- or His-fusion proteins, cDNAs of IFITM1 and EphA2 were integrated into the pGEX6p-1-GST/His vector. EphA2, gB and gH/L overexpression vectors were gifted by Prof. Mu-Sheng Zeng and Hua Zhang (Sun Yat-sen University Cancer Center). For site-directed mutagenesis, cDNAs of wild-type IFITM1 (IFITM1^w^), IFITM1 with the Tyr-121 mutation IFITM1 (IFITM1^m1^), IFITM1 with the Leu-104 mutation IFITM1 (IFITM1^m2^) and IFITM1 with the Tyr-121 and Leu-104 mutations IFITM1 (IFITM1^m1+2^) were integrated into the pLVX-blast vector.

For plasmid transfection, NP69, HK1 or HEK293 cells were plated onto 6-well plates (1 × 10^5^ cells per well) 1 day before transfection; then the medium was replaced with fresh medium and cells were incubated for 1 h. The appropriate dosage of plasmid was mixed with lipofectamine 2000 and added to each well to ensure the same total dose. The wells were mixed by gentle shaking; then plates were incubated for 6–8 h. The medium was replaced with fresh medium and plates were incubated for another 40 h before subsequent experiments.

### EBV preparation and cell-free infection

Recombinant EBV (EBV-GFP) was generated from EBV-positive Burkitt lymphoma cells (EBV+Akata) and a cell-free infection was conducted^19,20^. EBV with GFP integrated was produced from Akata cells based on a previous study^19^ with slight modifications. Briefly, Akata-EBV cells were crosslinked with 0.8% (v/v) goat anti-human IgG (Bersee) for 6 h; then the cells were cultured for 72 h with standard medium. EBV particles were collected by centrifugation at 54,000 × *g* for 2 h and resuspended with serum-free DMEM. The virus suspension was aliquoted in 0.5 ml aliquots and stored at −80 °C or used immediately for infection studies. For cell-free infection, NP69, HK1 and HEK293 were exposed to EBV for 3 h before detecting the number of copies of the viral genome. For detection of the infection efficiency, cells were cultured for an additional 72 h, after which the medium was replaced. The infection efficiency was roughly judged under a fluorescence microscope (Leica) and precisely measured using a flow cytometer (Spectral Cell Analyzer, Sony) on the basis of GFP expression.

### Antiviral test of sIFITM1 in nude mice

Female BALB/c nude mice aged 6–8 weeks were randomly distributed into four distinct groups (eight per group). Sample size was determined according to previous virus experiments in vivo. We assumed that data distribution was normal although without a formal test. All mice subjected to different stimuli were maintained in the same environmental conditions for growth. Female mice were housed in groups of four, with 12 h light and 12 h dark conditions, feeding on a standard diet. The room temperature was 22 °C and humidity was 50%. Two groups were xenografted with HEK293 cells and the other two groups were xenografted with vector or IFITM1-overexpressing HEK293 cells subcutaneously. After epithelial-cell clusters formed and were detectable, sIFITM1 (5 μg each time for one mouse) or the sIFITM1 solvent (H_2_O) was injected at the subcutaneous injection site of HEK293 cells 3 times at 10 h intervals, and EBV-GFP was injected (5 × 10^6^ IU each time for one mouse) 3 times at 10 h intervals after the first injection of sIFITM1/H_2_O (Fig. 1k). After 30 h, the mice were euthanized and epithelial-cell clusters were collected for the detection of EBV copy numbers by *Taq*Man−qPCR.

### RNA isolation, reverse transcription and RT−qPCR

Total RNA extraction was performed using RNA Trizol (Invitrogen). RNA was transcribed into cDNA using the All-in-One First-Strand cDNA Synthesis kit (TransGen) following manufacturer instructions. The real-time qPCR mixture was prepared following manufacturer instructions using the PerfectStart Green qPCR SuperMix kit supplemented with Dye II (Transgen), and the reaction was run on an ABI Prism 7500 (ABI). The expression level of each mRNA was normalized to the housekeeping gene B_2_M mRNA level and the fold change relative to the control represented the expression difference. The RT−qPCR primers (5ʹ to 3ʹ) are listed below:

IFITM1-F: CATCCGGAAGAAACTGGT

IFITM1-R: TCCCACAAAGCCAACTC

EphA2-F: CCCGATGAGATCACCGTCAG

EphA2-R: GGCACCGATATCCTGGAAGG

YTHDF1-F: ACCTGTCCAGCTATTACCCG

YTHDF1-R: TGGTGAGGTATGGAATCGGAG

YTHDF2-F: AGCCCCACTTCCTACCAGATG

YTHDF2-R: TGAGAACTGTTATTTCCCCATGC

YTHDF3-F: TCAGAGTAACAGCTATCCACCA

YTHDF3-R: GGTTGTCAGATATGGCATAGGCT

B_2_M-F: TGAAGCTGACAGCATTCGG

B_2_M-R: CTGCTGGATGACGTGAGTAAA

### Western blotting

Cell lysates were collected in RIPA buffer (Thermo Fisher, 89900) or cell lysis buffer and supplemented with a commercial protease inhibitor cocktail (Thermo Fisher, 78442) before use. Total protein was obtained by centrifugation and the protein concentration was determined by a Pierce BCA Protein Assay kit (Thermo Fisher, 23225). An equal amount of total protein was fractionated by 8～12% SDS–PAGE and transferred onto a 0.22 μm PVDF membrane (Millipore, ISEQ 00010). Membranes were immunoblotted with the indicated primary antibodies and then incubated with HRP-conjugated secondary antibody. Primary antibodies were as follows: mouse anti-IFITM1 (60074-1, Proteintech, 5B5E2, KD/KO validated, 1/1,000), rabbit anti-EphA2 (6997, CST, 1/1,000), rabbit anti-EGFR (18986-1, Proteintech, KD/KO validated, 1/1,000), rabbit anti-DDX5 (ab126730, Abcam, EPR7239, KD/KO validated, 1/1,000), rabbit anti-DDX6 (ab174277, Abcam, EPR12146, KD/KO validated, 1/1,000), rabbit anti-DDX17 (ab180190, Abcam, EPR13807(B), KD/KO validated, 1/1,000), rabbit anti-Actin (YT0096, ImmunoWay, 1/5,000) and rabbit anti-GAPDH (ab9485, Abcam, 1/5,000). Secondary antibodies were HRP-conjugated goat anti-rabbit IgG (SA00001-2, Proteintech, 1/5,000) and HRP-conjugated goat anti-mouse IgG (SA00001-1, Proteintech, 1/5,000). Blots were incubated with ECL substrate (BioRad, 1705061) and imaged with the ECL detection system (ChemiDoc, BioRad).

### Flow cytometry

To determine EBV infection rates, 1 × 10^6^ cells incubated with EBV were collected and washed using 1×PBS containing 0.2% bovine serum albumin (BSA). Cells were then resuspended in 300 μl of 1×PBS containing 0.2% BSA. Data were acquired using an LE-SA3800 Spectral Analyzer (Sony) and FlowJo software was used for analysis.

### Indirect immunofluorescence assay

To detect the co-localization of IFITM1 and EphA2 or IFITM1 and EGFR, cells were plated on glass-bottom cell culture plates (801006, NEST) for 24 h. After brief washing twice with 1×PBS, cells were fixed with 4% paraformaldehyde (BioSharp) and permeabilized with 0.2% Triton X-100 (Thermo Fisher, 89900). Plates were washed gently and blocked with 3% normal goat serum (C0265, Beyotime). Subsequently, the cells were incubated with the primary antibody pairs overnight, followed by incubation with respective fluorophore-conjugated secondary antibodies (Alexa Fluor 488 goat anti-mouse IgG H&L and Alexa Fluor 647 goat anti-rabbit IgG H&L; 150113, 150079, Abcam) for 1 h and counterstained with DAPI (C1005, Beyotime) for 10 min at room temperature. To avoid false-positive cross-reactivity, primary antibody pairs were chosen from different species (mouse anti-IFITM1 and rabbit anti-EphA2, mouse anti-IFITM1 and rabbit anti-EGFR). Fluorescence images were recorded using the High Content Analysis System (CQ1, Yokogawa).

### Co-immunoprecipitation

Immunoprecipitation was performed using an IP kit (abs955, Absin) following supplier instructions. Briefly, whole-cell proteins were isolated in lysis buffer and the lysate was centrifuged; the supernatant was subsequently collected. To lower the background, 500 µl of supernatant containing ~500–1,000 µg protein was incubated with 5 µl of protein A and 5 µl of protein G for 1 h at 4 °C. Total protein lysates that removed unspecific binding proteins were obtained after centrifugation. For protein binding, 1–5 µg of the corresponding antibodies (to IFITM1 or EphA2, as mentioned above) and homologous IgG (eBiosciences) was added to pre-cleaned protein lysates. Immediately afterwards, samples were incubated at 4 °C overnight under gentle rotation. To precipitate the target proteins, 5 µl of protein A and 5 µl of protein G were added to bind the antigen–antibody complexes; the reaction was maintained at 4 °C for 3 h. After gently washing three times with wash buffer, the unbound proteins were removed and the pellet (agarose–antibody–antigen complex) was resolved in 20–40 µl of SDS-loading buffer for further western blotting with the indicated antibodies. For exogenous co-IP of IFITM1, EphA2, gH/gL and gB, HEK293 cells were transfected with the corresponding combination of Myc-gH/gL, Myc-gB, pSMCV-IFITM1, pSMCV-EphA2 and empty vector (see figure legends for details).

### Protein expression and purification

IFITM1, gB, gH/gL and EphA2 DNAs were constructed into pGEX6p-1-GST/His vector and recombinant fusion proteins were purified in the Rosetta strain of *E. coli*. Briefly, after the constructed plasmids were cloned into pET28a vector, *E. coli* Rosetta star pLysS cells were transformed with these plasmids. Colonies were inoculated into 25 ml of Luria-Bertani (LB) media containing ampicillin (100 μg ml^−1^) and grown for 16 h at 37 °C; then 25 ml cultures were transferred into 500 ml of fresh LB medium and grown at 37 °C for 4 h, adding isopropyl-β-d-thiogalactopyranoside (IPTG, 1 mM) to continue cultivation for 24 h at 16 °C. Bacterial cells were collected by centrifugation and 6×His- or GST-tagged target proteins were purified through His-tag or GST-tag affinity chromatography (Qiagen) and desalted using an Amersham column.

### Competitive binding assays

First, 96-well microtitre plates were coated with 200 ng of GST-EphA2 overnight at 4 °C, followed by blocking with 5% BSA in 1×PBS for 2 h at room temperature. Then, gradient concentrations of 6×His-IFITM1 and 6×His-gB or 6×His-gH/gL proteins were added and incubated for 2 h at room temperature. After washing with 1×BST (0.05% Tween-20 in 1×PBS), the plate was incubated with a rabbit anti-GST antibody or mouse anti-His antibody (1:4,000 dilution) for 2 h at room temperature. The plate was then washed and incubated at 37 °C for 1 h with HRP-conjugated goat anti-rabbit antibody or HRP-conjugated goat anti-mouse antibody. After adding the substrate tetramethyl-benzidine, the absorbance was measured at 450 nm using the Nanodrop One spectrophotometer (Thermo Fisher).

### 3D structure and spatial binding site prediction

Two main methods, I-TASSER from the University of Michigan and SWISS-model from the European Center for Bioinformatics, were used to select the model consistent with the local structure published in ref. ^41^. After selecting a single predicted structure, the 3D structures of the complex formed by IFITM1, EphA2 and gH/gL were calculated. The top 100 models were selected from the 2,000 rigid docking models optimized by geometry and electrostatics. On the basis of the accurately predicted binding sites and side-chain flexibility of the binding interface amino acids, 200,000 models were generated, and the 10 best models were selected after clustering and scoring. After comparing with the model published in ref. ^41^, the first model was selected for subsequent calculations.

### Site-specific mutation

IFITM1 mutant primers were designed by the homologous recombination method, and IFITM1 wild-type plasmids were amplified by PCR using mutant-specific primers and the KOD-Plus-Neo amplification kit (Toyobo). After mutation, the PCR products were recombined at 37 °C for 30 min. The top 10 competent cells were added to the recombinant products, and the transformation mixture was evenly spread onto an LB plate containing ampicillin and incubated overnight at 37 °C. Single clones were selected for Sanger sequencing.

### MeRIP-seq and MeRIP−qPCR

m^6^A RNA immunoprecipitation was performed according to the Magna MeRIP m^6^A kit (17-10499, Merck) instructions. Total RNA was extracted by the Trizol method and then interrupted to form fragments of ~100 nt. Beads with anti-m^6^A antibodies were added and incubated at 4 °C for 8 h to form m^6^A RNA–antibody–magnetic bead complexes, which were adsorbed using a magnetic rack and washed several times to remove impurities. The m^6^A RNA was eluted by competitive binding and submitted for sequence analysis by RT−qPCR.

### RIP-seq and RIP−qPCR

RNA immunoprecipitation (RIP) was performed according to the instructions of the Magna RIP RNA-Binding Protein Immunoprecipitation kit (Merck). Cells lysed by lysis buffer were incubated with anti-YTHDF3 antibody and magnetic beads at 4 °C for 8 h. The magnetic bead–antibody–target protein–RNA complex was adsorbed with a magnetic rack and cleaned 5 times with wash buffer to remove impurities. RNA was extracted by Trizol, and the purified RNA was analysed by RT−qPCR or submitted for sequence analysis.

### Tandem affinity purification pull-down and mass spectrometry

Wide-type full-length YTHDF3 and its double m^6^A binding site defective mutants, designated YTHDF3^WT^ and YTHDF3^DM^, were constructed into a tandem affinity purification vector pLVpuro-TAP (SBP-3HA). Then, three stably expressing TAP-Vector, TAP-YTHDF3^WT^ OE and TAP-YTHDF3^DM^ OE HK1 cell lines were subjected to 2 µg ml^−1^ puromycin selection for a week. The TAP affinities of the exogenously expressed TAP-Vector, -YTHDF3^WT^ and -YTHDF3^DM^ cells were extracted from the three cell lysates by excess streptavidin resin; then the endogenously expressed YTHDF3 partners were immunoprecipitated by YTHDF3 antibodies coupled to protein A/G beads. Finally, the YTHDF3 exogenously expressed TAP affinities and endogenously expressed immunoprecipitates were separately identified by mass spectrometry.

### RNA stability assay

To assess IFITM1 RNA stability, cells were incubated with actinomycin (ActD) to terminate transcription. Briefly, HK1 cells were incubated with ActD (5 μg ml^−1^) for 0, 30 and 60 min, and collected. Total RNA was extracted and the IFITM1 RNA expression was determined by RT−qPCR.

### Statistical analyses

Data are presented as mean ± s.e.m. derived from at least three independent experiments. All statistical analyses, including Pearson’s correlation coefficients (*r*), *t*-tests and so on were two-tailed and executed using GraphPad Prism 8. Although the data conformed to the assumptions of the statistical tests utilized, normality of data distribution was presumed but was not rigorously tested. In addition, the collection and analysis of data were carried out in a blinded manner relative to experimental conditions and no animal or data point was excluded from the analysis for any reason.

### Reporting summary

Further information on research design is available in the Nature Portfolio Reporting Summary linked to this article.

### Supplementary information

Reporting Summary

### Source data

Source Data Fig. 1 Statistical source data.

Source Data Fig. 2 Unprocessed western blots and statistical source data.

Source Data Fig. 3 Statistical source data.

Source Data Fig. 4 Unprocessed western blots and statistical source data.

Source Data Fig. 5 Statistical source data.

Source Data Fig. 6 Unprocessed western blots and statistical source data.

Source Data Extended Data Fig. 1 Unprocessed western blots and statistical source data.

Source Data Extended Data Fig. 2 Unprocessed western blots and statistical source data.

Source Data Extended Data Fig. 3 Unprocessed western blots and statistical source data.

Source Data Extended Data Fig. 4 Statistical source data.

Source Data Extended Data Fig. 5 Unprocessed western blots.

Source Data Extended Data Fig. 6 Unprocessed western blots and statistical source data.

Source Data Extended Data Fig. 7 Unprocessed western blots.

Source Data Extended Data Fig. 8 Statistical source data.

Source Data Extended Data Fig. 9 Unprocessed western blots and statistical source data.

Source Data Extended Data Fig. 10 Statistical source data.

## Data Availability

The datasets that support the findings of this study are available within the paper. Transcriptomic datasets generated in this study can be found on the NCBI Sequence Read Archive (SRA) under BioProject PRJNA946546 and PRJNA976759. The prediction of the protein–protein interactions was performed using the STRING database (http://string-db.org/). The prediction of the three-dimensional structure of IFITM1, EphA2 and gH/gL was performed using I-TASSER (https://zhanglab.ccmb.med.umich.edu/I-TASSER/) and SWISS-model (https://swissmodel.expasy.org/). The raw sequencing data of the MeRIP-seq can be found under PRJNA997768. Mass spectrometry datasets can be accessed via Harvard Dataverse at 10.7910/DVN/QHCEZI (ref. ^57^). Source data are provided with this paper.
