# Supplementary material for: Interferon-induced transmembrane protein-1 competitively blocks Ephrin receptor A2-mediated Epstein–Barr virus entry into epithelial cells
Source: Nat Microbiol. 2024 Apr 22;9(5):1256–70. doi: 10.1038/s41564-024-01659-0 (PMC11087256; doi:10.1038/s41564-024-01659-0)
Supplement: Supplementary file 1 — Reporting Summary [file 41564_2024_1659_MOESM1_ESM.pdf]

nature portfolio

Corresponding author(s): Xin Li

Last updated by author(s): Feb 4, 2024

Reporting Summary

Nature Portfolio wishes to improve the reproducibility of the work that we publish. This form provides structure for consistency and transparency in reporting. For further information on Nature Portfolio policies, see our Editorial Policies and the Editorial Policy Checklist.

Statistics

For all statistical analyses, confirm that the following items are present in the figure legend, table legend, main text, or Methods section.

n/a	Confirmed	
☐	☒	The exact sample size (*n*) for each experimental group/condition, given as a discrete number and unit of measurement
☐	☒	A statement on whether measurements were taken from distinct samples or whether the same sample was measured repeatedly
☐	☒	The statistical test(s) used AND whether they are one- or two-sided<br>*Only common tests should be described solely by name; describe more complex techniques in the Methods section.*
☒	☐	A description of all covariates tested
☒	☐	A description of any assumptions or corrections, such as tests of normality and adjustment for multiple comparisons
☐	☒	A full description of the statistical parameters including central tendency (e.g. means) or other basic estimates (e.g. regression coefficient) AND variation (e.g. standard deviation) or associated estimates of uncertainty (e.g. confidence intervals)
☐	☒	For null hypothesis testing, the test statistic (e.g. *F*, *t*, *r*) with confidence intervals, effect sizes, degrees of freedom and *P* value noted<br>*Give P values as exact values whenever suitable.*
☒	☐	For Bayesian analysis, information on the choice of priors and Markov chain Monte Carlo settings
☒	☐	For hierarchical and complex designs, identification of the appropriate level for tests and full reporting of outcomes
☐	☒	Estimates of effect sizes (e.g. Cohen's *d*, Pearson's *r*), indicating how they were calculated

Our web collection on statistics for biologists contains articles on many of the points above.

Software and code

Policy information about availability of computer code

Data collection	We didn’t use software for data collection in this study.
Data analysis	The following software were used for data analysis: GraphPad Prism 8, FlowJo VX.

For manuscripts utilizing custom algorithms or software that are central to the research but not yet described in published literature, software must be made available to editors and reviewers. We strongly encourage code deposition in a community repository (e.g. GitHub). See the Nature Portfolio guidelines for submitting code & software for further information.

Data

Policy information about availability of data

All manuscripts must include a data availability statement. This statement should provide the following information, where applicable:

- Accession codes, unique identifiers, or web links for publicly available datasets
- A description of any restrictions on data availability
- For clinical datasets or third party data, please ensure that the statement adheres to our policy

The datasets that support the findings of this study are available within the paper and Supplementary Information. Transcriptomic datasets generated in this study can be found on the NCBI Sequence Read Archive (SRA) under BioProject PRJNA946546 and PRJNA976759. Predication of the protein–protein interactions was performed at STRING database (http://string-db.org/). The predication of three-dimensional structure of IFITM1, EphA2 and gH/gL was performed using I-TASSER (https://zhanglab.ccmb.med.umich.edu/I-TASSER/) and SWISS-model (https://swissmodel.expasy.org/). The raw sequencing data of the MeRIP-seq can be found

under PRJNA997768. Mass spectrometry datasets can be accessed at https://doi.org/10.7910/DVN/QHCEZI. The source data underlying Figures, Extended Data Figures and Supplementary Figures are provided as a Source Data file.

Human research participants

Policy information about studies involving human research participants and Sex and Gender in Research.

Reporting on sex and gender	In our study, the clinical sample size was relatively smaller and the selection of clinical samples was conduct on a random basis. As a result, we did not explicitly stratify for sex and/or gender. We believe that this is unlikely to impact the validity of our analysis results.
Population characteristics	All patients were diagnosed with NPC according to the 2017 edition for staging of nasopharyngeal carcinoma in China and were aged between 30 and 69 years. The age range for normal controls was 30-52 years.
Recruitment	Not applicable.
Ethics oversight	This study complies with all relevant ethical regulations approving by the Medical Ethics Committee of Southern Medical University.

Note that full information on the approval of the study protocol must also be provided in the manuscript.

Field-specific reporting

Please select the one below that is the best fit for your research. If you are not sure, read the appropriate sections before making your selection.

☒ Life sciences ☐ Behavioural & social sciences ☐ Ecological, evolutionary & environmental sciences

For a reference copy of the document with all sections, see nature.com/documents/nr-reporting-summary-flat.pdf

Life sciences study design

All studies must disclose on these points even when the disclosure is negative.

Sample size	We use a relatively smaller sample size in our study. Although we do not use statistical methods to predetermine the sample size, our sample size is consistent with what has been reported in other previous nasopharyngeal carcinoma studies, such as an article published in Oncotarget, titled `Up-regulation of long non-coding RNA AFAP1-AS1 expression is associated with progression and poor prognosis of nasopharyngeal carcinoma.`
Data exclusions	No data were excluded from the analysis.
Replication	The experiments were biologically replicated at least three times independently.
Randomization	In our study, we used randomization in the mouse experiments; animals in the experimental and control groups were randomly assigned. We also considered the randomization in clinical samples.
Blinding	The collection and analysis of data were conducted blindly to the experimental conditions.

Reporting for specific materials, systems and methods

We require information from authors about some types of materials, experimental systems and methods used in many studies. Here, indicate whether each material, system or method listed is relevant to your study. If you are not sure if a list item applies to your research, read the appropriate section before selecting a response.

Materials & experimental systems

n/a	Involved in the study	
☐	☒	Antibodies
☐	☒	Eukaryotic cell lines
☒	☐	Palaeontology and archaeology
☐	☒	Animals and other organisms
☒	☐	Clinical data
☒	☐	Dual use research of concern

Methods

n/a	Involved in the study	
☐	☒	ChIP-seq
☐	☒	Flow cytometry
☒	☐	MRI-based neuroimaging

Antibodies

Antibodies used	mouse anti-IFITM1 (#60074-1, Proteintech, 5B5E2, KD/KO VALIDATED, 1/1000), rabbit anti-EphA2 (#6997, CST, 1/1000), rabbit anti-DDX5(#ab126730, Abcam, EPR7239, KD/KO VALIDATED, 1/1000), rabbit anti-DDX6 (#ab174277, Abcam, EPR12146, KD/KO VALIDATED, 1/1000), rabbit anti-DDX17(#ab180190, Abcam, EPR13807(B), KD/KO VALIDATED, 1/1000), rabbit anti-ACTIN (#YT0096, ImmunoWay, 1/5000), and rabbit anti-GAPDH (#ab9485, Abcam, 1/5000), HRP-conjugated goat anti-rabbit IgG (#SA00001-2, Proteintech, 1/5000) and HRP-conjugated goat anti-mouse IgG (#SA00001-1, Proteintech, 1/5000).
Validation	Validation of commercial antibodies and target specificity was confirmed in the technical data sheets provided by the manufacture, containing example data and relevant citations.1.mouse anti-IFITM1 (Proteintech#60074-1): Validated. Details provided by the manufacture:https://www.ptglab.co.jp/Products/IFITM1-Antibody-60074-1-Ig.htm#protocols. 2.rabbit anti-EphA2 (CST#6997): Details provided by the manufacture: https://www.cellsignal.com/products/primary-antibodies/epha2-d4a2-xp-rabbit-mab/6997. 3. rabbit anti-DDX5 (Abcam#ab126730): Validated. Details provided by the manufacture: https://www.abcam.com/en-au/products/primary-antibodies/ddx5-antibody-epr7239-ab126730. 4. rabbit anti-DDX6 (Abcam#ab174277): Validated. Details provided by the manufacture: https://www.abcam.cn/products/primary-antibodies/ddx6-antibody-epr12146-ab174277.html 5. rabbit anti-DDX17(Abcam#ab180190): Validated. Details provided by the manufacture: https://www.abcam.com/en-hk/products/primary-antibodies/ddx17-antibody-epr13807b-ab180190

Eukaryotic cell lines

Policy information about cell lines and Sex and Gender in Research

Cell line source(s)	The cell lines include NP460 , NP69, NP460-EBV, HK1, HK1-EBV, C666-1, Akata, AGS, HEK293 and Daudi, all cell lines are human. NP460 , NP69, NP460-EBV, HK1, HK1-EBV, C666-1 and Akata cells were kindly provided by Professor Sai-Wah Tsao's group (The University of Hong Kong, Pokfulam, Hong Kong SAR, China). AGS and HEK293 cells were maintained in our laboratory. Daudi cells were purchased from FuHeng Cell Center (Shanghai).
Authentication	All cells underwent STR analyses. Addtionally, we did not use any cross-contaminated cell lines based on the lists provided by International Cell Line Authentication Committee(ICLAC).
Mycoplasma contamination	All cells underwent mycoplasma tests (Myco-Blue ® Mycoplasma detector, Vazyme) that showed negative.
Commonly misidentified lines (See ICLAC register)	No commonly misidentified cell lines were used in the study based on ICLAC.

Animals and other research organisms

Policy information about studies involving animals; ARRIVE guidelines recommended for reporting animal research, and Sex and Gender in Research

Laboratory animals	The animals used in this study were female BALB/c nude mice, aged 6-8 weeks.
Wild animals	No wild animals were used in the study.
Reporting on sex	As usual, all mice were female.
Field-collected samples	The mouse housing conditions comprised a standard 12-hour light/dark cycle, with an ambient temperature maintained between 20-22°C and a relative humidity level of 40-60%.
Ethics oversight	Animal experiments were conducted following protocols approved by the Medicine Animal Care and Use Guidelines of Southern Medical University .

Note that full information on the approval of the study protocol must also be provided in the manuscript.

Flow Cytometry

Plots

Confirm that:

☒ The axis labels state the marker and fluorochrome used (e.g. CD4-FITC).

☒ The axis scales are clearly visible. Include numbers along axes only for bottom left plot of group (a 'group' is an analysis of identical markers).

☒ All plots are contour plots with outliers or pseudocolor plots.

☒ A numerical value for number of cells or percentage (with statistics) is provided.

Methodology

Sample preparation	To determine EBV infection rates, 1 × 10^6 cells incubated with EBV were collected and washed using 1×PBS containing 1%

Sample preparation	bovine serum albumin (BSA). Cells were then resuspended in 300 µL of 1×PBS containing 1% BSA.
Instrument	Data were acquired using an LE-SA3800 Spectral Analyzer (Sony).
Software	FlowJo VX software was used for analysis.
Cell population abundance	1 × 10^6 cells incubated with EBV were collected for each sample.
Gating strategy	We set the gates according to scattered light, which are commonly referred as forward scatter (FSC) and side scatter (SSC). The size of FSC was positively correlated with cell diameter; the larger the cell was, the larger the FSC was. The smaller the vice versa. The size of SSC was positively correlated with the mass of intracellular granular structure. For different cells, the more complex the intracellular granular structure was, the larger the mass was, and the larger the SSC was. The smaller the vice versa.

☒ Tick this box to confirm that a figure exemplifying the gating strategy is provided in the Supplementary Information.
