# Supplementary figures and images for: Interferon-induced transmembrane protein-1 competitively blocks Ephrin receptor A2-mediated Epstein–Barr virus entry into epithelial cells
Source: Nat Microbiol. 2024 Apr 22;9(5):1256–70. doi: 10.1038/s41564-024-01659-0 (PMC11087256; doi:10.1038/s41564-024-01659-0)

**b**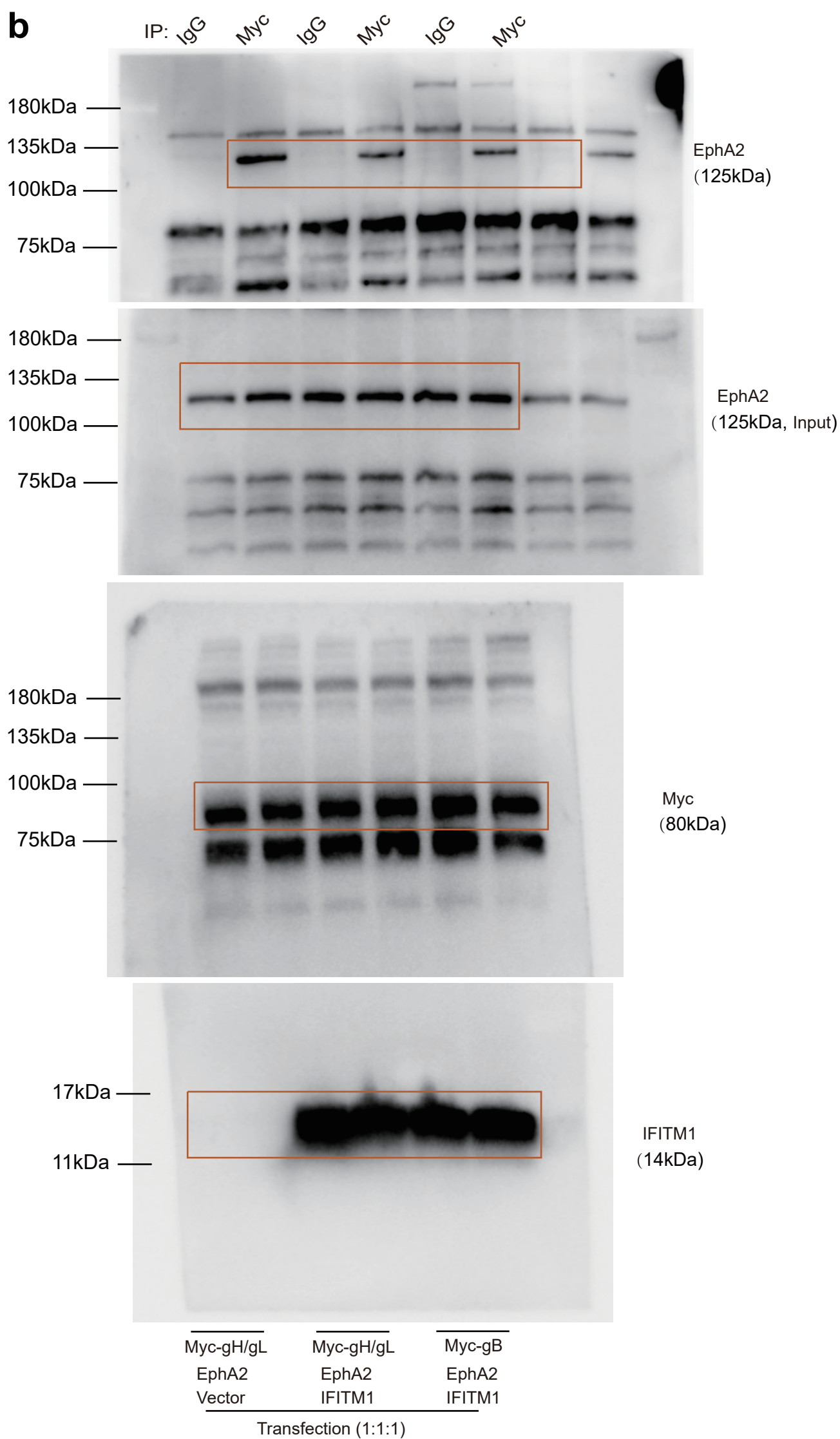

Supplement: Supplementary file 3 — Unprocessed western blots and statistical source data. [file 41564_2024_1659_MOESM3_ESM.zip › SourceData_Fig2/SourceData_Fig2b_left.pdf.pdf]

**b**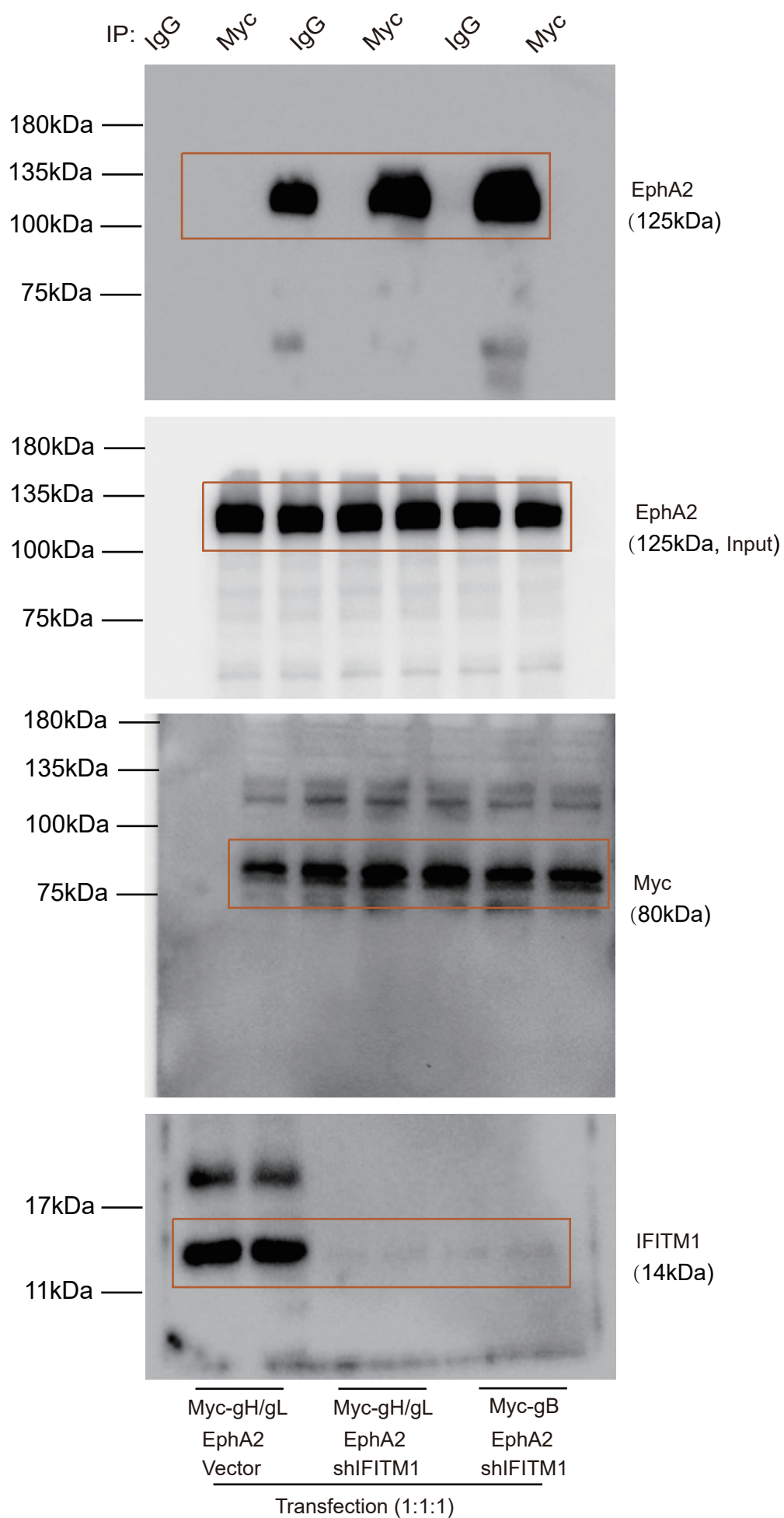

Supplement: Supplementary file 3 — Unprocessed western blots and statistical source data. [file 41564_2024_1659_MOESM3_ESM.zip › SourceData_Fig2/SourceData_Fig2b_right.pdf.pdf]

C

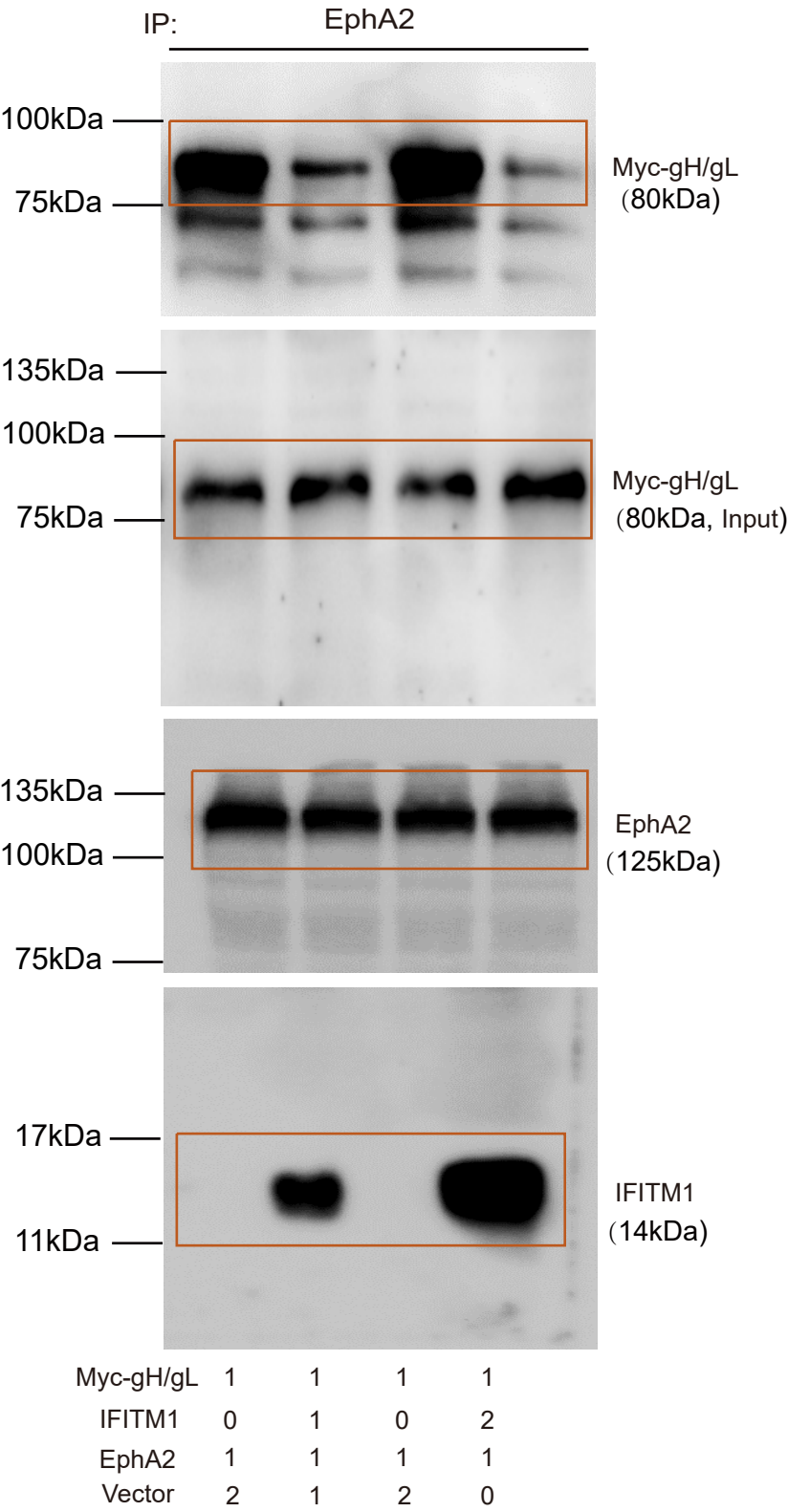

Supplement: Supplementary file 3 — Unprocessed western blots and statistical source data. [file 41564_2024_1659_MOESM3_ESM.zip › SourceData_Fig2/SourceData_Fig2c.pdf.pdf]

**f**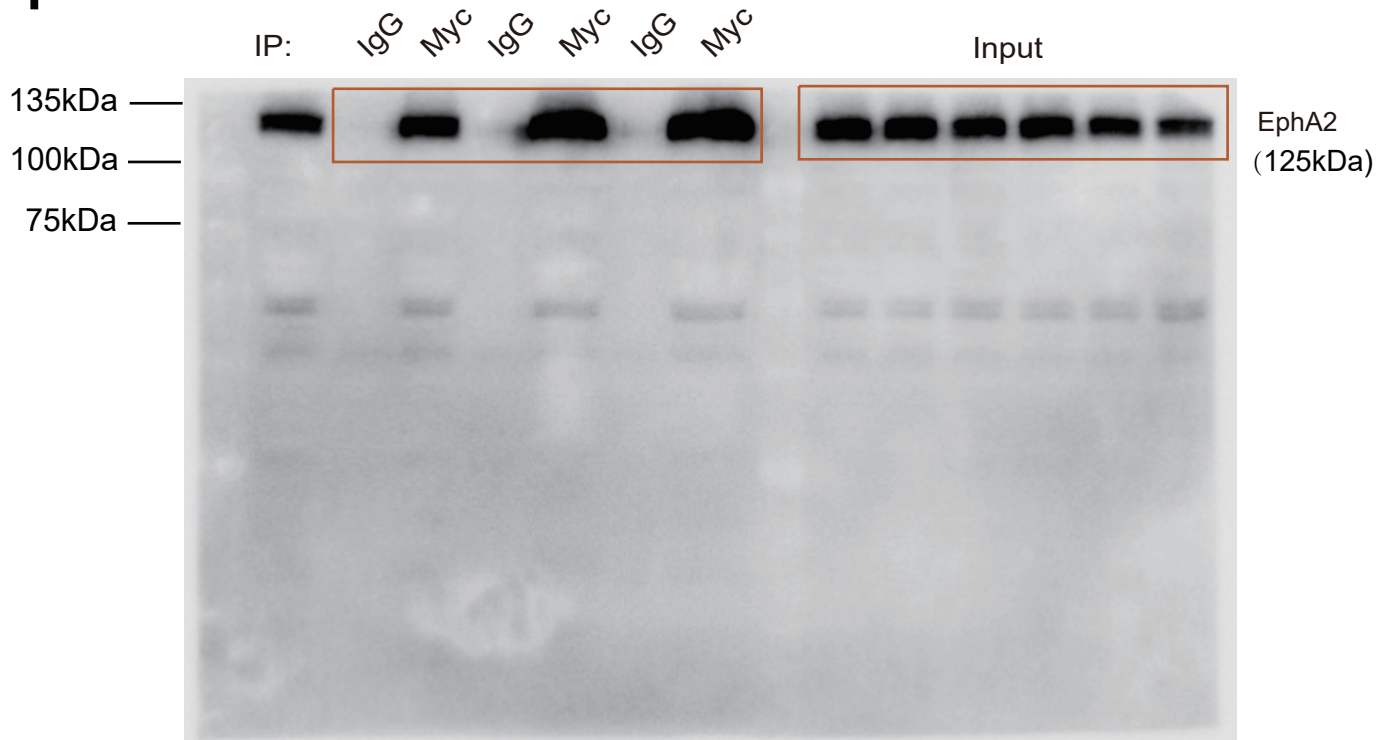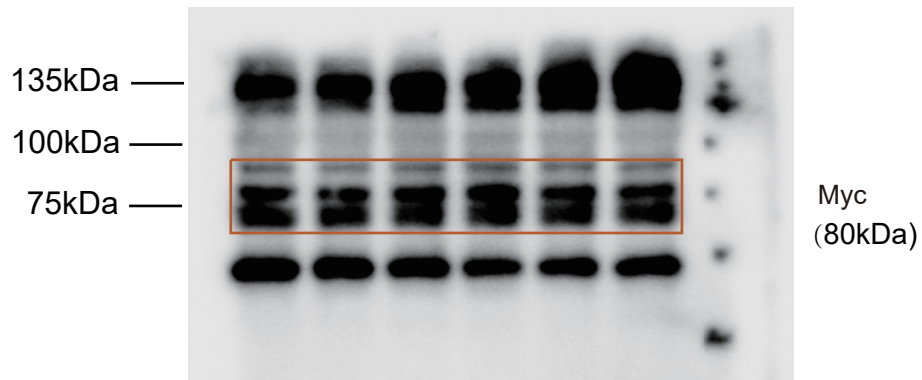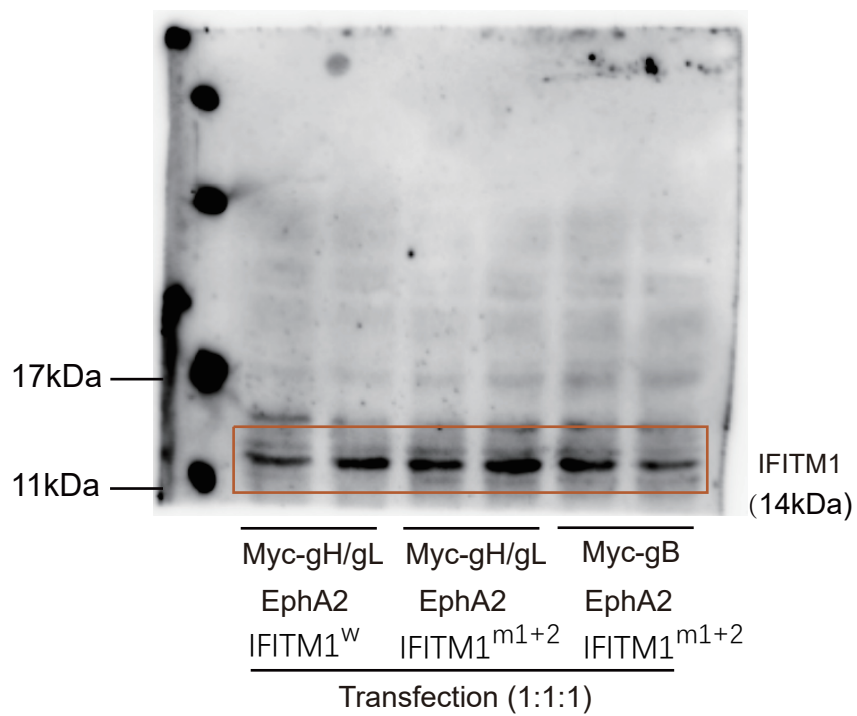

Supplement: Supplementary file 5 — Unprocessed western blots and statistical source data. [file 41564_2024_1659_MOESM5_ESM.zip › SourceData_Fig4/SourceData_Fig4f.pdf.pdf]

**C**

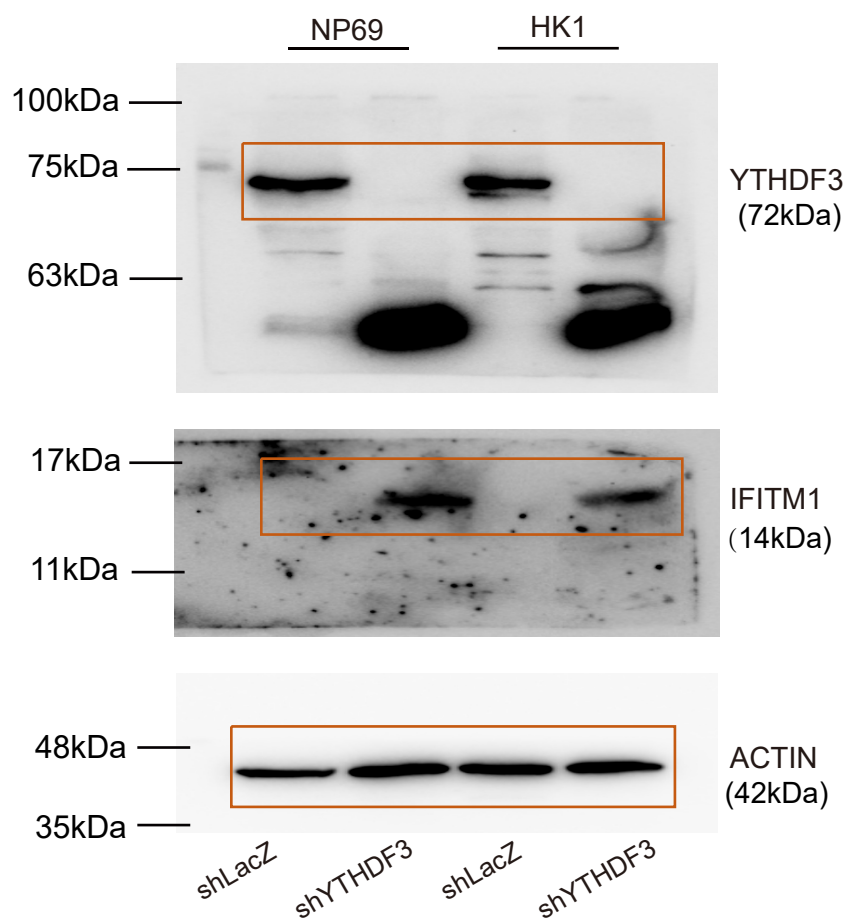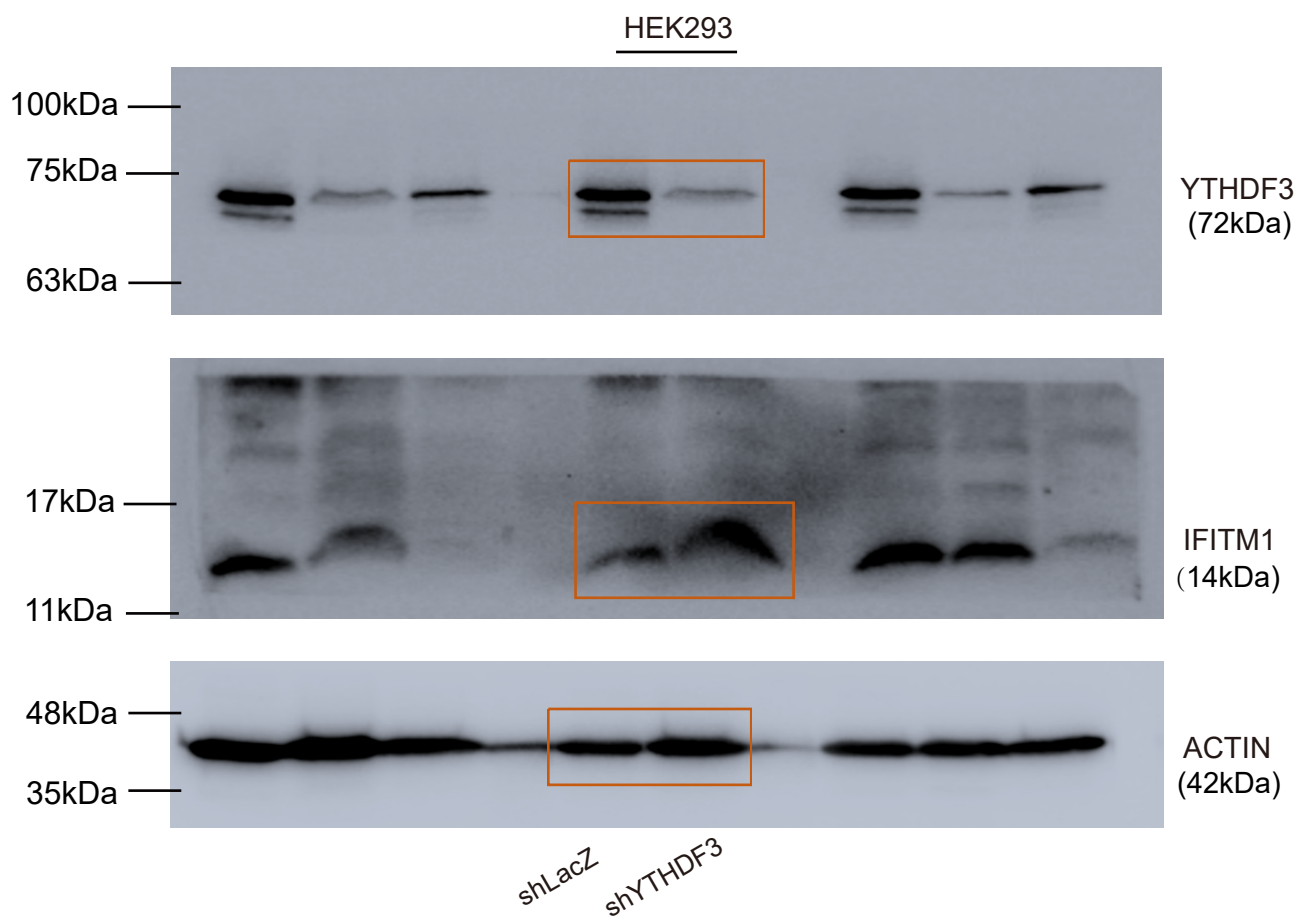

Supplement: Supplementary file 6 — Statistical source data. [file 41564_2024_1659_MOESM6_ESM.zip › SourceData_Fig5/SourceData_Fig5c.pdf.pdf]

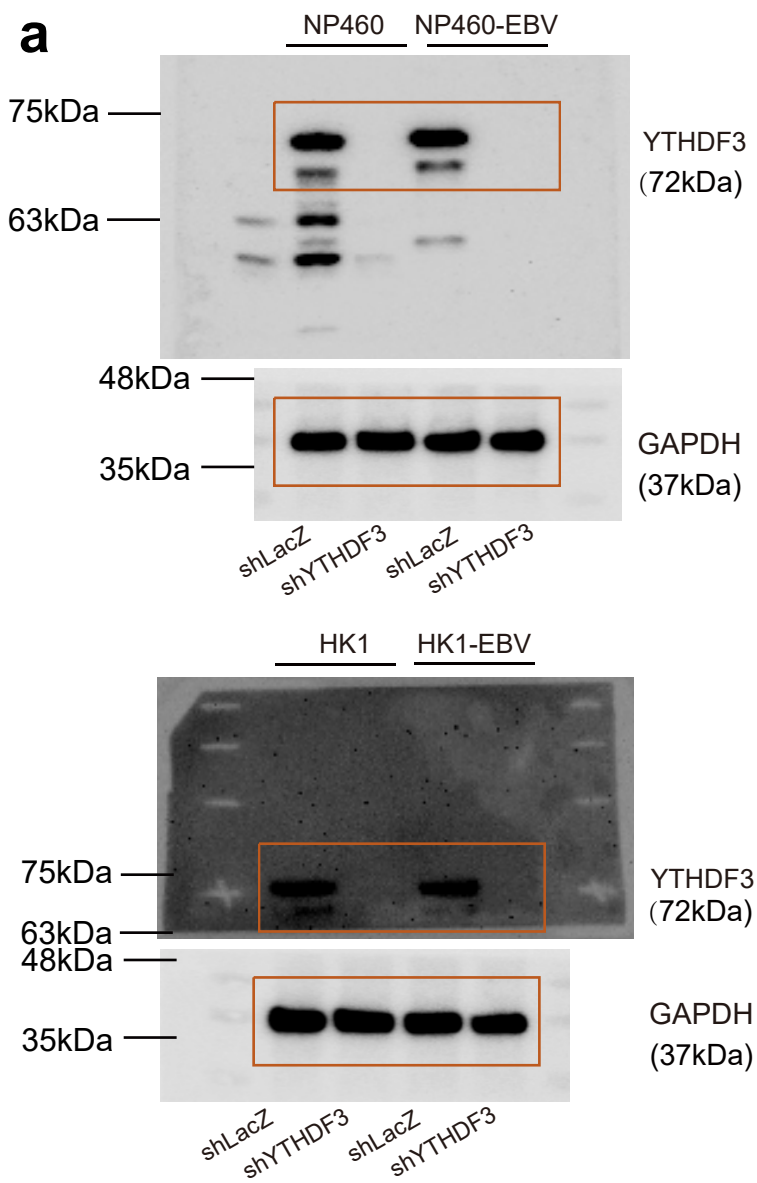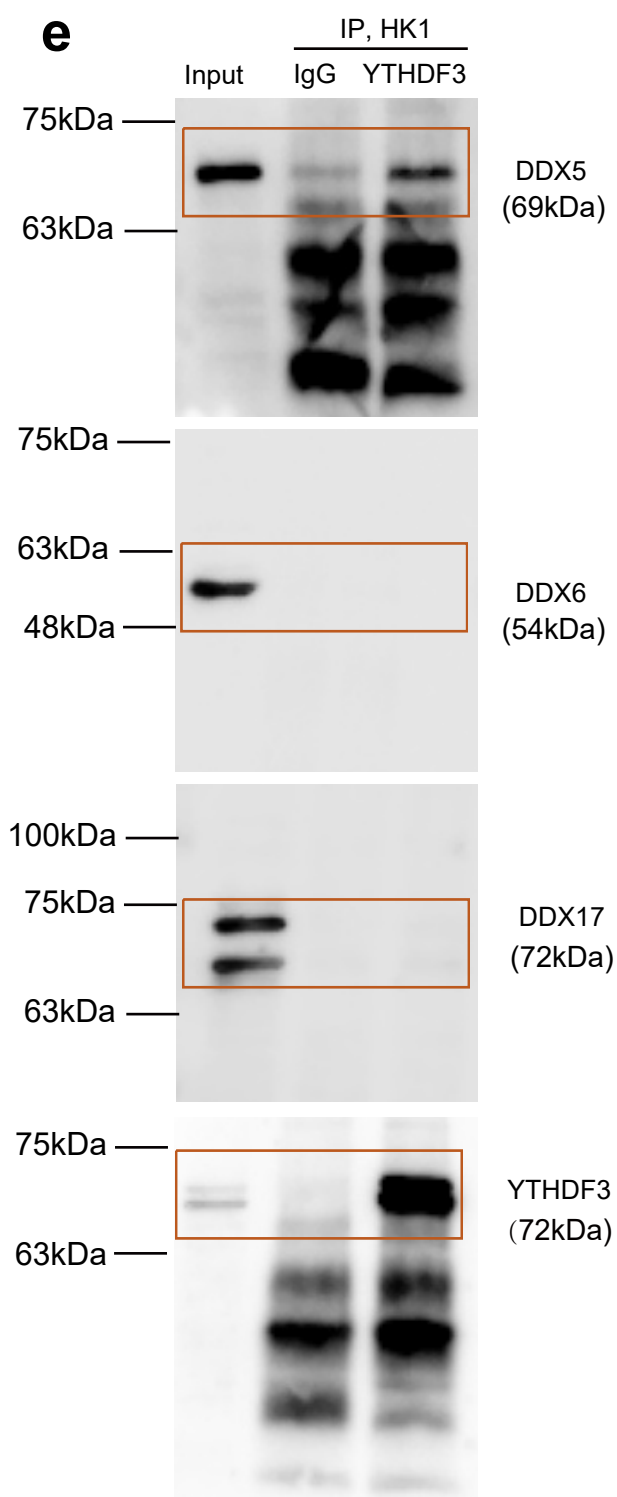

Supplement: Supplementary file 7 — Unprocessed western blots and statistical source data. [file 41564_2024_1659_MOESM7_ESM.zip › SourceData_Fig6/SourceData_Fig6a,e.pdf.pdf]

**f**

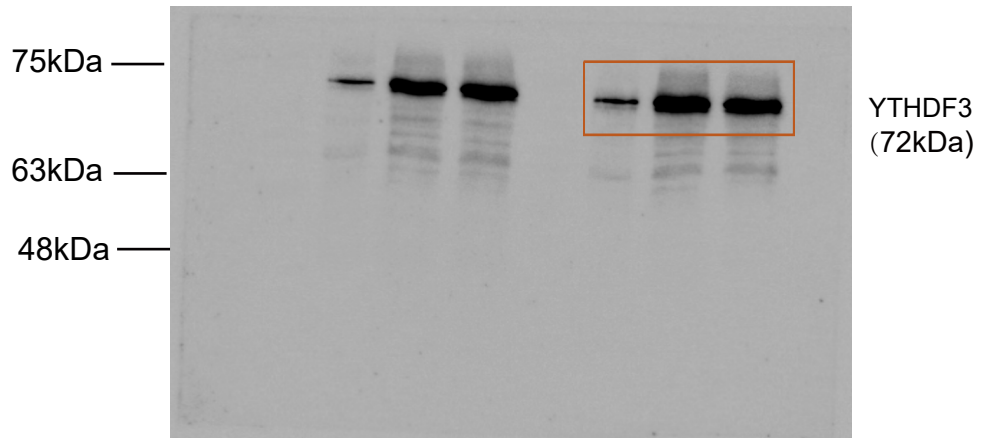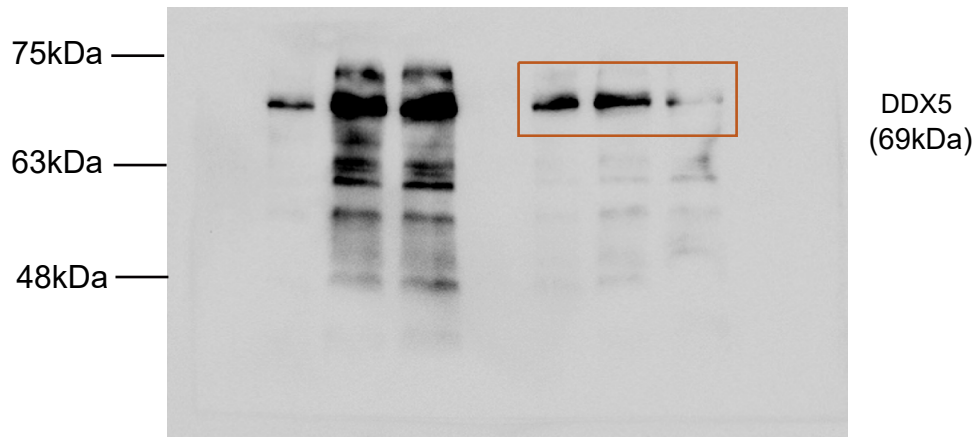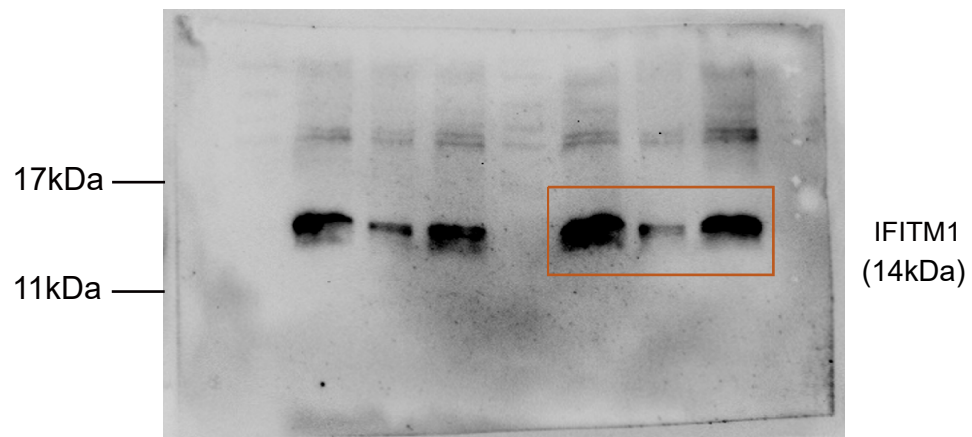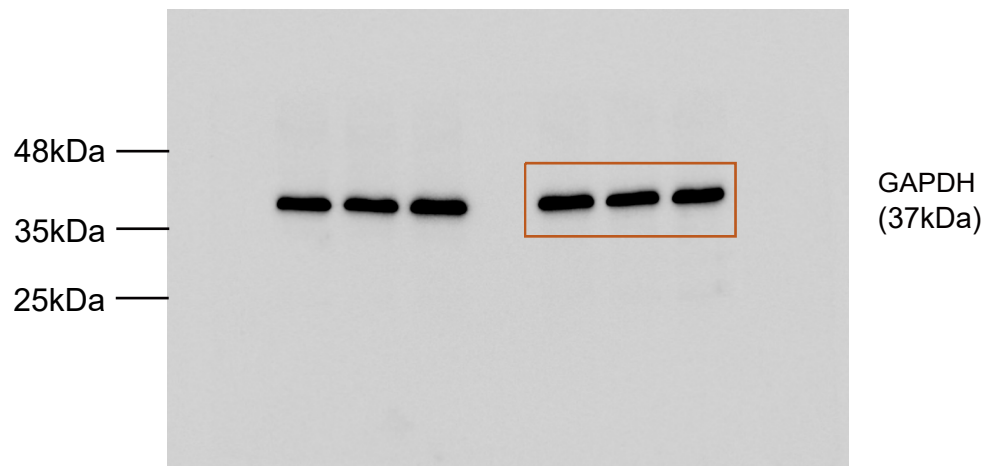

Vector+shLacZ  
YTHDF3 OE+shLacZ  
YTHDF3 OE+shDDX5

Supplement: Supplementary file 7 — Unprocessed western blots and statistical source data. [file 41564_2024_1659_MOESM7_ESM.zip › SourceData_Fig6/SourceData_Fig6f.pdf.pdf]

**C**

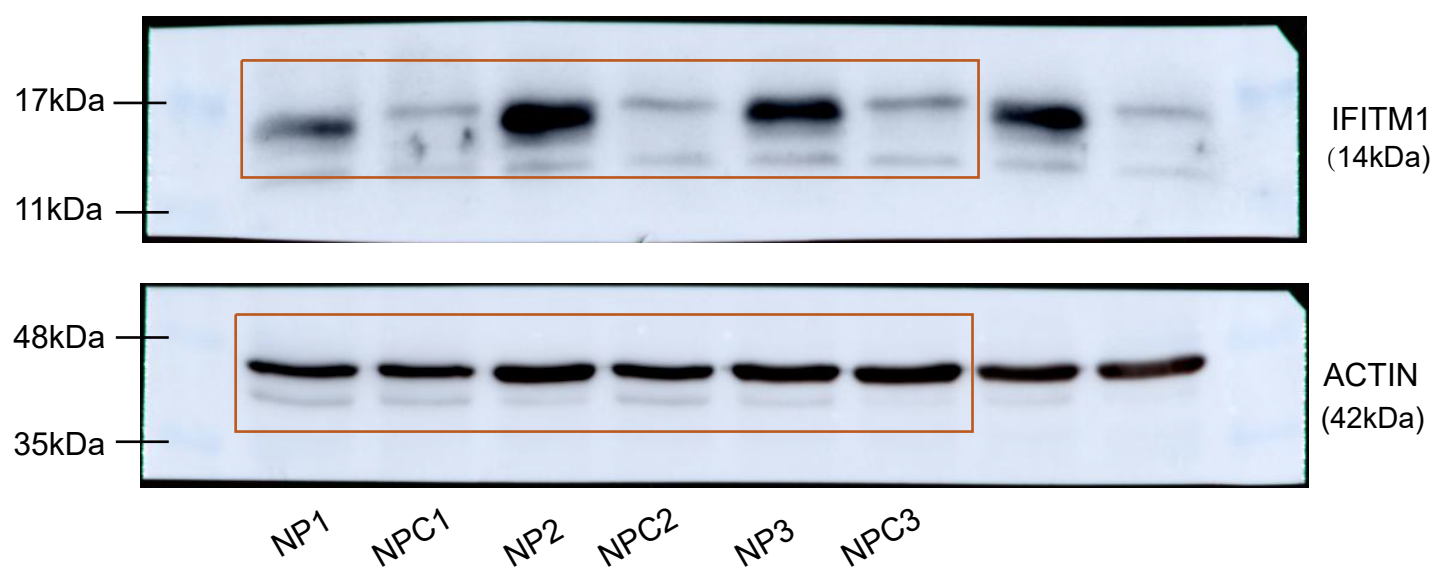

Supplement: Supplementary file 8 — Unprocessed western blots and statistical source data. [file 41564_2024_1659_MOESM8_ESM.zip › SourceData_ED_Fig1/SourceData_ED_Fig1c.pdf.pdf]

**a**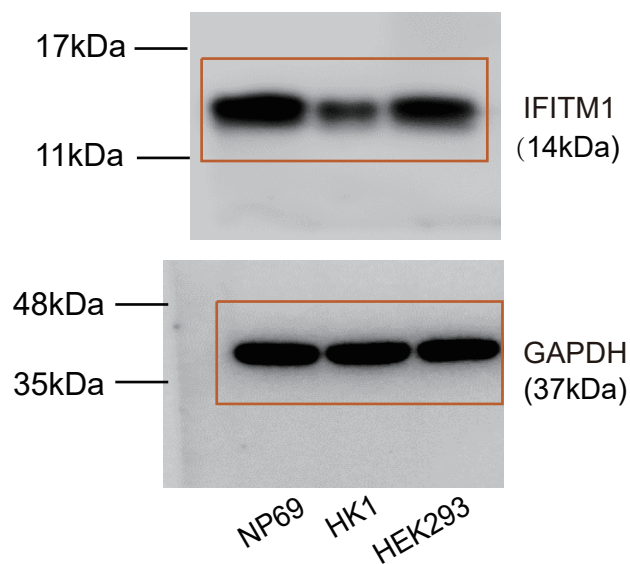**c**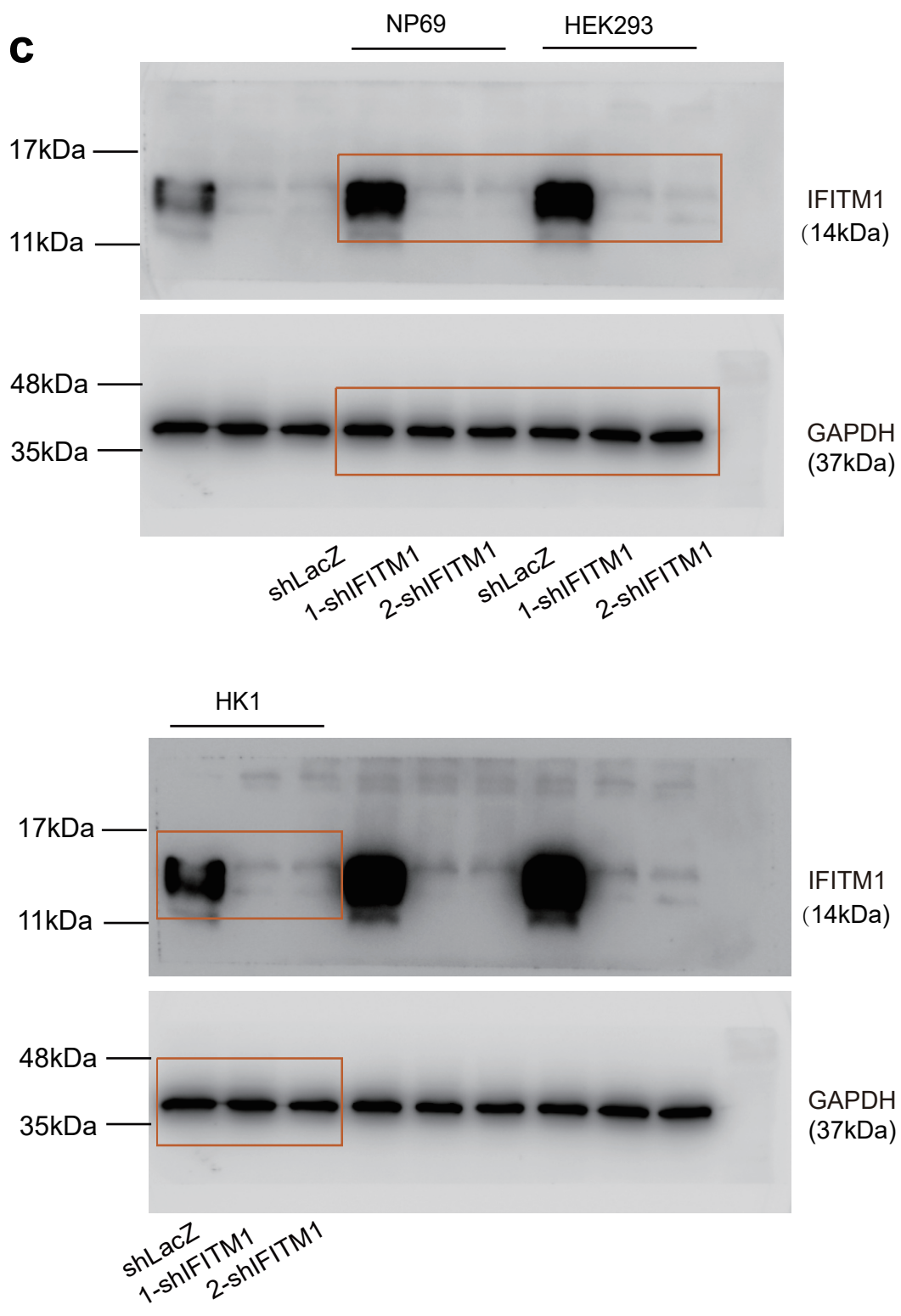

Supplement: Supplementary file 9 — Unprocessed western blots and statistical source data. [file 41564_2024_1659_MOESM9_ESM.zip › SourceData_ED_Fig2/SourceData_ED_Fig2a,c.pdf.pdf]

**b**

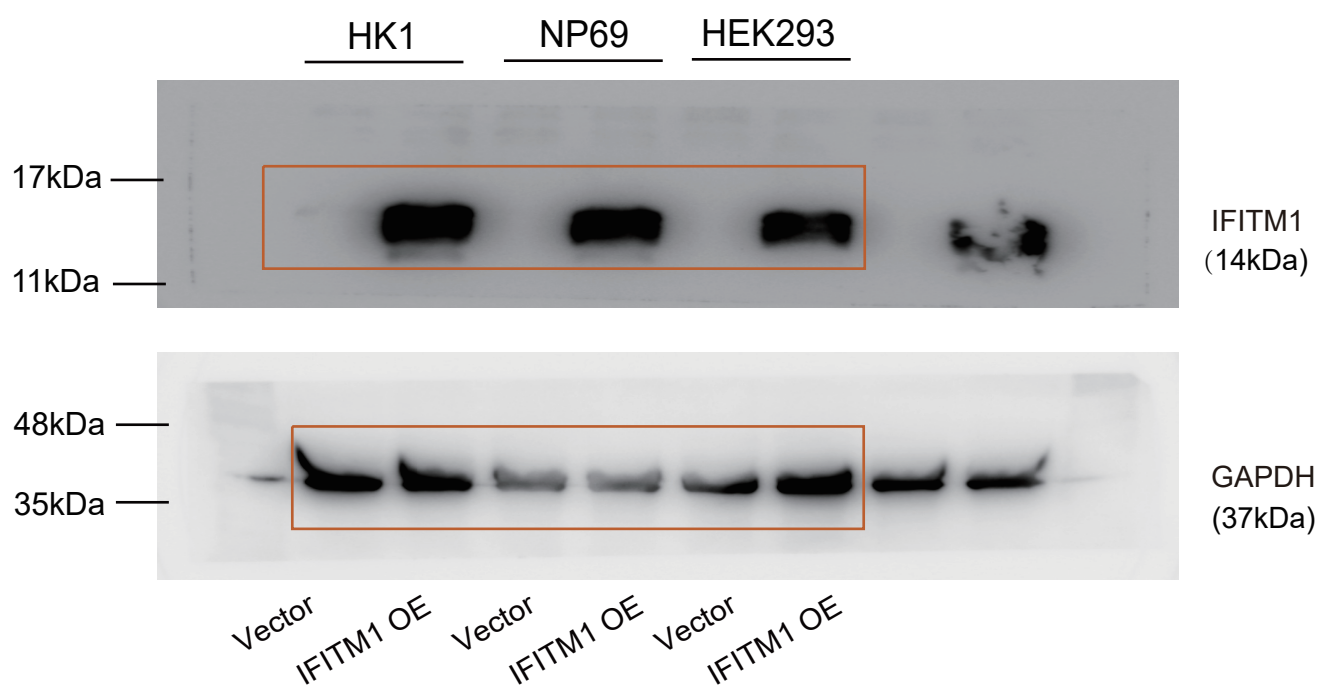

Supplement: Supplementary file 10 — Unprocessed western blots and statistical source data. [file 41564_2024_1659_MOESM10_ESM.zip › SourceData_ED_Fig3/SourceData_ED_Fig3b.pdf.pdf]

**b**

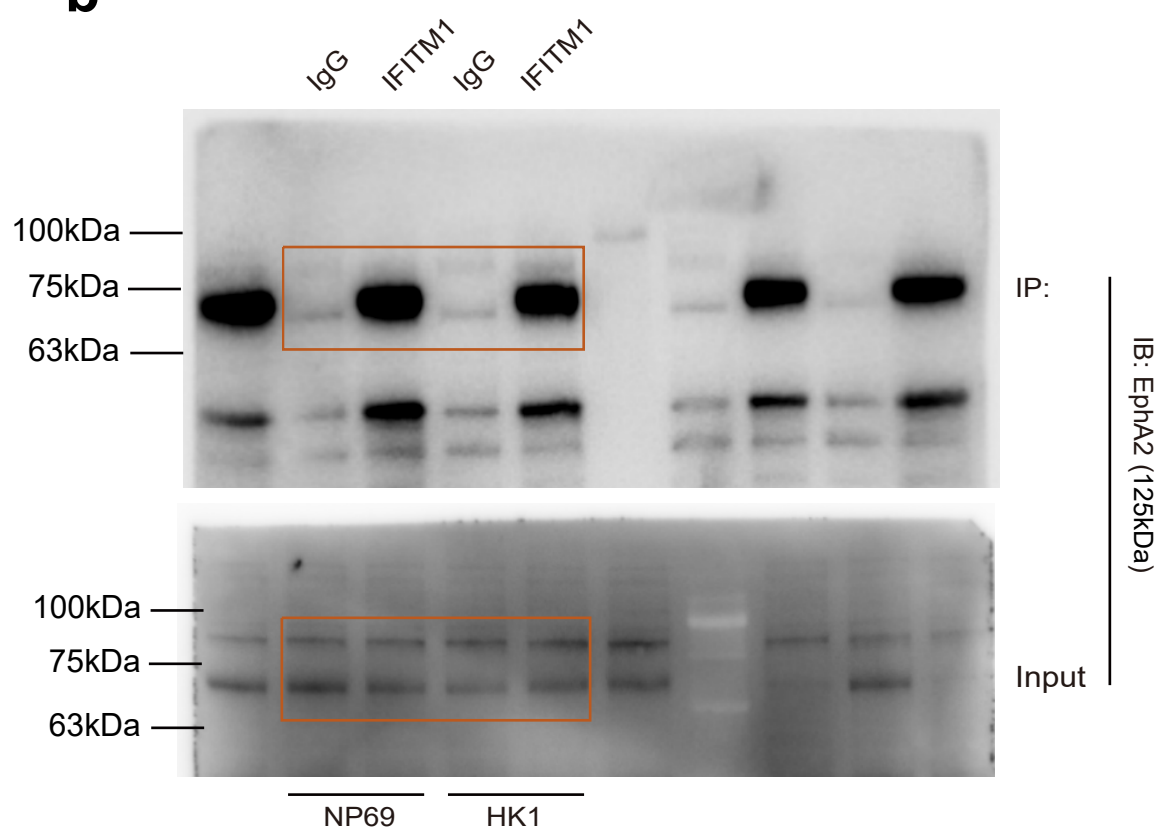

Supplement: Supplementary file 12 — Unprocessed western blots. [file 41564_2024_1659_MOESM12_ESM.zip › SourceData_ED_Fig5/SourceData_ED_Fig5b.pdf.pdf]

**C**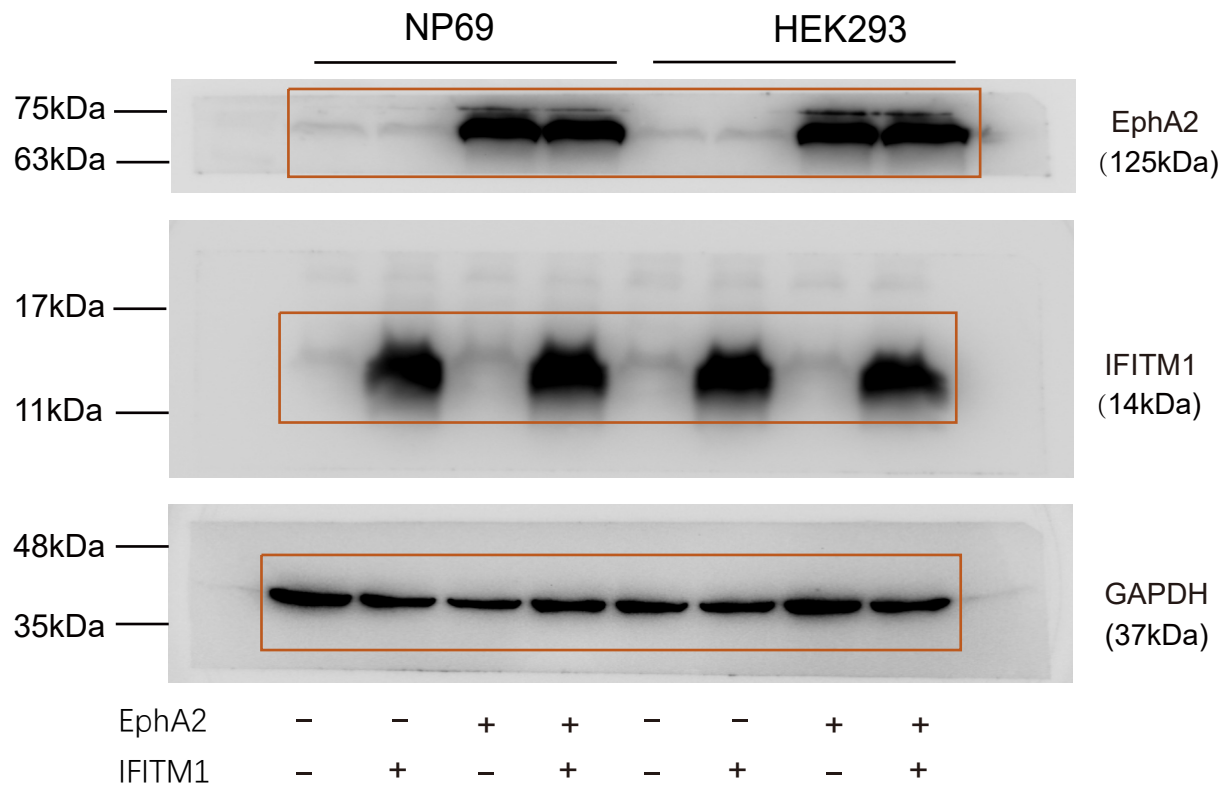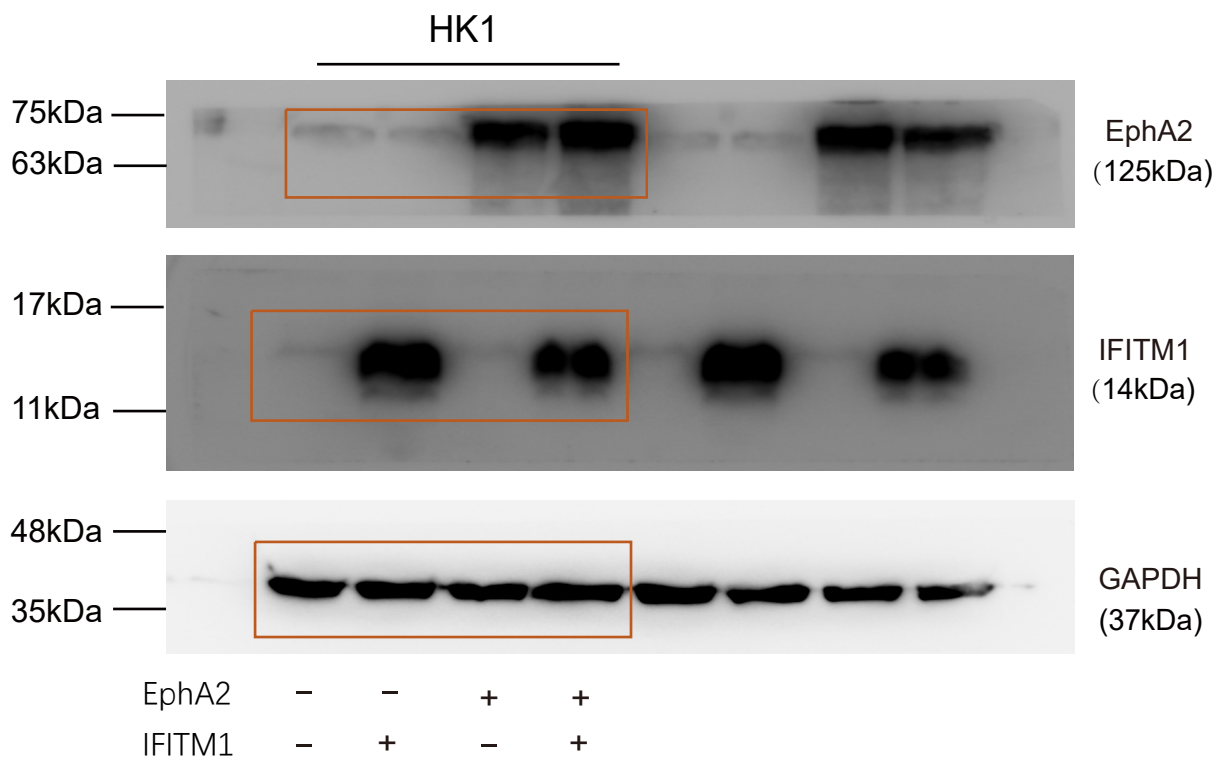

Supplement: Supplementary file 13 — Unprocessed western blots and statistical source data. [file 41564_2024_1659_MOESM13_ESM.zip › SourceData_ED_Fig6/SourceData_ED_Fig6c.pdf.pdf]

**C**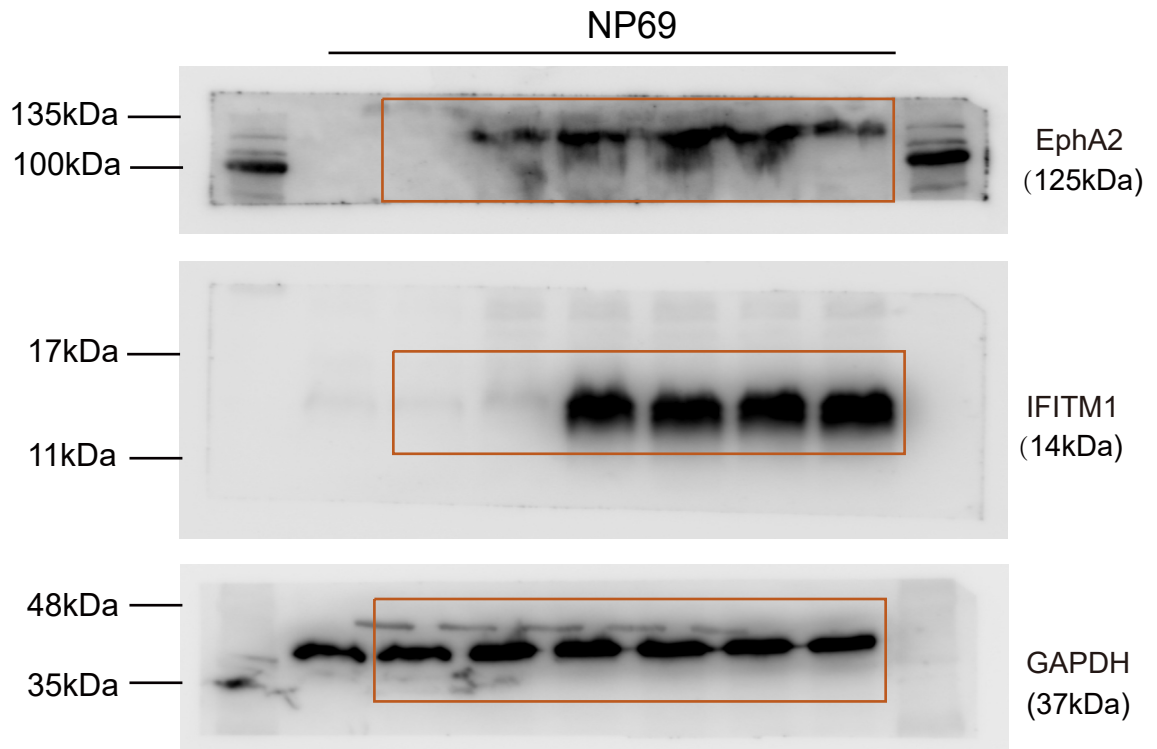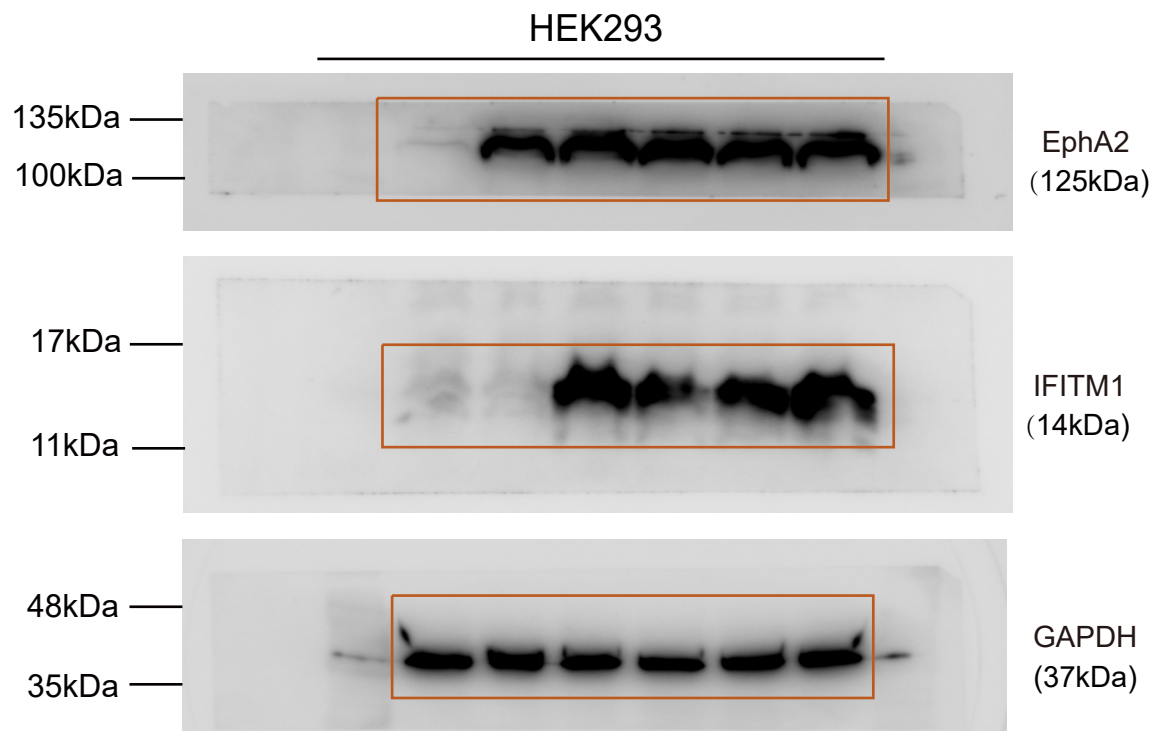

|                        |   |   |   |   |   |   |
|------------------------|---|---|---|---|---|---|
| EphA2                  | - | + | + | + | + | + |
| IFITM1 <sup>w</sup>    | - | - | + | - | - | - |
| IFITM1 <sup>m1</sup>   | - | - | - | + | - | - |
| IFITM1 <sup>m2</sup>   | - | - | - | - | + | - |
| IFITM1 <sup>m1+2</sup> | - | - | - | - | - | + |

Supplement: Supplementary file 14 — Unprocessed western blots. [file 41564_2024_1659_MOESM14_ESM.zip › SourceData_ED_Fig7/SourceData_ED_Fig7c.pdf.pdf]

**d**

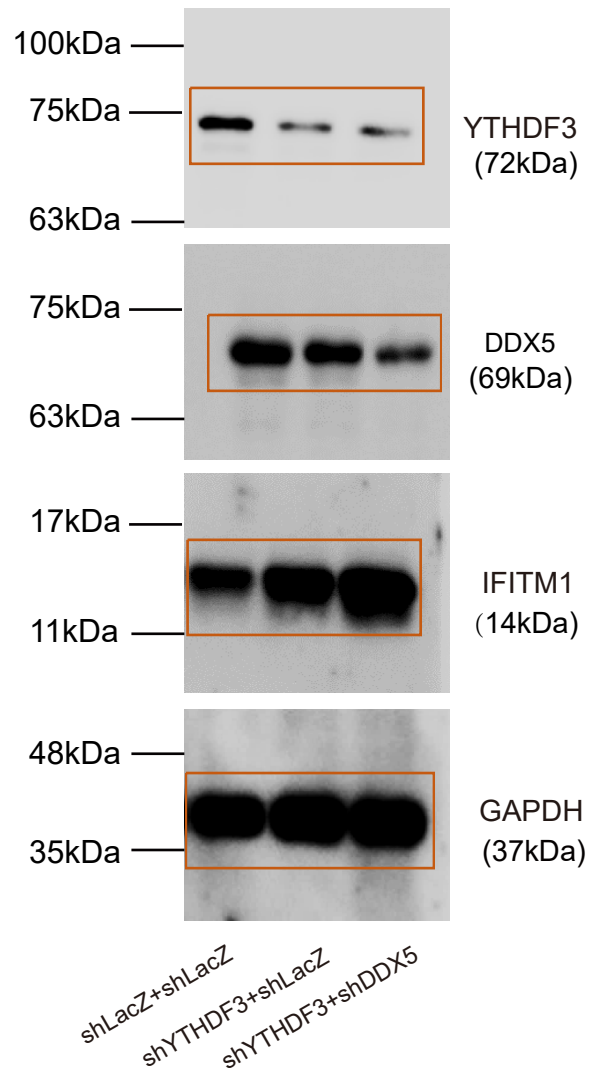

Supplement: Supplementary file 16 — Unprocessed western blots and statistical source data. [file 41564_2024_1659_MOESM16_ESM.zip › SourceData_ED_Fig9/SourceData_ED_Fig9d.pdf.pdf]
